# Constitutively active microglial populations limit anorexia induced by the food contaminant deoxynivalenol

**DOI:** 10.1186/s12974-022-02631-7

**Published:** 2022-11-19

**Authors:** Stéphanie Gaige, Rym Barbouche, Manon Barbot, Sarah Boularand, Michel Dallaporta, Anne Abysique, Jean-Denis Troadec

**Affiliations:** 1 Aix-Marseille University, CNRS, Laboratoire de Neurosciences Cognitives, UMR 7291, 3 Place Victor Hugo, 13331 Marseille, France; 2 Aix-Marseille University, CNRS, Centrale Marseille, FSCM (FR1739), PRATIM, 13397 Marseille, France

**Keywords:** Mycotoxin, Hypothalamus, Brainstem, Circumventricular organs, IBA1, TMEM119, CD68, CD11b, PLX3397, Minocycline

## Abstract

Microglia are involved in neuroinflammatory processes during diverse pathophysiological conditions. To date, the possible contribution of these cells to deoxynivalenol (DON)-induced brain inflammation and anorexia has not yet been evaluated. DON, one of the most abundant trichothecenes found in cereals, has been implicated in mycotoxicosis in both humans and farm animals. DON-induced toxicity is characterized by reduced food intake, weight gain, and immunological effects. We previously showed that exposure to DON induces an inflammatory response within the hypothalamus and dorsal vagal complex (DVC) which contributes to DON-induced anorexia. Here, in response to anorectic DON doses, we reported microglial activation within two circumventricular organs (CVOs), the area postrema (AP) and median eminence (ME) located in the DVC and the hypothalamus, respectively. Interestingly, this microglial activation was observed while DON-induced anorexia was ongoing (i.e., 3 and 6 h after DON administration). Next, we took advantage of pharmacological microglia deletion using PLX3397, a colony-stimulating factor 1 receptor (CSF1R)-inhibitor. Surprisingly, microglia-depleted mice exhibited an increased sensitivity to DON since non-anorectic DON doses reduced food intake in PLX3397-treated mice. Moreover, low DON doses induced c-Fos expression within feeding behavior-associated structures in PLX3397-treated mice but not in control mice. In parallel, we have highlighted heterogeneity in the phenotype of microglial cells present in and around the AP and ME of control animals. In these areas, microglial subpopulations expressed IBA1, TMEM119, CD11b and CD68 to varying degrees. In addition, a CD68 positive subpopulation showed, under resting conditions, a noticeable phagocytotic/endocytotic activity. We observed that DON strongly reduced CD68 in the hypothalamus and DVC. Finally, inactivation of constitutively active microglia by intraperitoneal administration of minocycline resulted in anorexia with a DON dose ineffective in control mice. Taken together, these results strongly suggest that various populations of microglial cells residing in and around the CVOs are maintained in a functionally active state even under physiological conditions. We propose that these microglial cell populations are attempting to protect the brain parenchyma from hazardous molecules coming from the blood. This study could contribute to a better understanding of how microglia respond to environmental contaminants.

**Supplementary Information:**

The online version contains supplementary material available at 10.1186/s12974-022-02631-7.

## Introduction

The control of body weight is a daily challenge and the ever-increasing proportion of people suffering from a nutritional imbalance illustrates this difficulty. Therefore, a deeper comprehension of the systems governing energy balance is still required. Complex elements that affect both the intake and expenditure sides of the energy balance equation are involved in the regulation of body weight. The brain is known for its essential role in regulating food intake via the control of hunger feeling and glucose homeostasis. Two central structures, i.e., the hypothalamus and the brainstem, strongly contribute to the homeostatic control of the energy balance by integrating information linked to nutritional status and arising from peripheral organs (gut, liver, pancreas, adipose tissue) [1]. Microglia cells are found throughout the brain and spinal cord, accounting for 5–20% of the total glial cell population in the central nervous system (CNS) parenchyma. These cells are known as "brain's immune cells", which sense extracellular cues and play important roles in the brain homeostasis, development, injuries, and recovery. Resting microglia cells characterized by small soma associated with long motile cellular processes continuously inspect the brain parenchyma and detect disruption of brain homeostasis [2]. When microglia detect a modification, they enter an active state characterized by morphological changes, taking on an amoeboid form with retracted processes and high mobility [3]. This dynamic mobility is considered an important first step in detecting local changes and triggering pattern-specific responses [4]. It should be mentioned here that in addition to ramified and amoeboid forms, several intermediate shapes have been described, including hypertrophic, dystrophic, and rod-shaped phenotypes [5, 6]. There is increasing evidence showing that microglia located in the brainstem and hypothalamus contribute to the regulation of energy balance. Microglia are positioned to sense and react to circulating signals that control energy metabolism and express receptors for a wide range of nutritional, hormonal, or immunological signals. They respond to a high-fat diet and regulate neurons functions to promote food intake and obesity in both the hypothalamus and brainstem [7–9].

Deoxynivalenol (DON) is the most common trichothecene toxin produced by fungi belonging to the *Fusarium* genus which commonly colonizes various cereals and represents a worldwide threat to agricultural production, food industries, and both animal and human health. An increasing number of countries are enacting restrictions or guidelines for DON contamination levels in food and feed [10]. The assessment of food contamination and human exposure revealed that a part of the world population is chronically exposed to DON concentrations near to or above the provisional maximum acceptable daily intake of 1 µg/kg body weight (BW) as defined by the joint Food and Agriculture Organization/World Health Organization Expert Committee on Food Additives [10]. In this context, children appear as population at high risk of dietary exposure to this mycotoxin due to their long-term ingestion of highly contaminated wheat flour and corn-based products [11–14]. The extensive occurrence of DON in human food is due to its stability throughout processing and heating [15, 16]. Given the wide human exposure to DON, investigations to improve our understanding of DON toxicity are still needed and should be carried out. Exposure to low or moderate DON doses induces a variety of effects in humans and animals including anorexia, vomiting, reduced weight gain, as well as neuroendocrine and immunological changes [17–21]. The mechanisms underlying DON-induced anorexia have not yet been fully elucidated, despite data revealing how it operates. After gavage or intraperitoneal (i.p.) injection, DON can acutely, i.e., 3 hours (h), increase the plasma levels of anorectic intestinal hormones such as cholecystokinin and glucagon like peptide-1 in mice, suggesting that these intestinal hormones contribute to the DON-induced anorexia [22]. On the other hand, vagotomy failed to attenuate DON-evoked c-Fos expression in the NTS [23], suggesting that the humoral pathway through CVOs of gut hormones released in response to DON or DON directly is privileged. In agreement, DON was shown to be rapidly distributed in various organs, including the brain, within a short time after exposure (~ 10 min) [24]. In line with this, anorexia brought on by per os (p.o.) intoxication may be reproduced by centrally injecting DON at dosages ineffective at the periphery. Moreover, centrally injected DON activated neurons that belong to central pathways strongly dedicated to the homeostatic control of food intake. These cell groups include pro-opiomelanocortin (POMC)-, tyrosine hydroxylase-, and nucleobindin-2 (NUCB2)/nesfatin-1-expressing neurons located both in key hypothalamic nuclei and the DVC [23–25]. Importantly, by performing meal pattern analysis, which allows the continuous study of ad libitum eating and provides a detailed description of the elements of feeding behavior, we previously revealed that *p.o.* DON decreased both meal frequency (satiety) and size (satiation). The observed effect on meal frequency suggested the presence of nausea-induced anorexia, but the effect on meal size and the activation of specific anorexic neuronal populations suggested modulation of meal termination mechanisms [23]. Interestingly, at moderate doses, DON toxicosis is characterized by activation of the innate immune system and the subsequent increase in pro-inflammatory cytokines expression [18]. Immune cell lines have been shown to be highly sensitive to DON and respond by upregulating pro-inflammatory cytokine expression in vitro [18, 26]. Several studies have shown an up-regulation of pro-inflammatory cytokines: interleukin-1β (IL-1β), interleukin-6 (IL-6), and tumor necrosis factor-α (TNF-α) in peripheral organs such as the spleen, liver, kidney, and small intestine [27, 28]. Interestingly, a direct link between DON-induced reduction in food intake and cytokines up-regulation after acute oral DON administration has been demonstrated [29, 30]. In accordance, DON intoxication enhances mRNA production of IL-1β, IL-6, and TNF-α in the hypothalamus and in the DVC, two central structures that act as gateways for circulating chemicals and are strongly associated with food intake regulation [31].

In this context, the present study sought to determine the contribution of microglial cells to DON-induced anorexia. For this, we focused our study on DON-induced anorexia under conditions of deletion and/or inhibition of microglial cells. Our results revealed an unexpected protective role of microglia against DON intoxication.

## Materials and methods

### Animal housing and treatments

Adult male C57BL/6J mice purchased from Charles River Laboratories (L’Abresle, France) were housed in standard cages, maintained in a controlled environment (12 h/12 h light–dark cycle, 22 °C and 40–50% humidity) with free access to water and food (A04 pellets, SAFE, Augy, France). PLX3397 was provided by ChemGood LLC (Glen Allen, USA) and formulated in A04 standard chow by SAFE (Augy, France) at 290 mg/kg chow as previously published [32]. Minocycline (M9511, Sigma) was administered intraperitoneally at 50 mg/kg BW, dissolved in 100 µl per 10 g BW of a physiological saline solution (0.9% NaCl) during three consecutive days. At the end of the third day, the animals received either a vehicle or DON (D-0156, Sigma Chemical Co.). To be accustomed to this i.p. administration, animals were handled and pricked daily using a 26 G needle in the same i.p. area for 3 days without chemical injection (minocycline or 0.9% NaCl).

#### *P.o.* DON administration

One hour before the beginning of the dark phase, the mice received orally 1.25 or 12.5 mg/kg BW DON, dissolved in 100 µl per 10 g BW of distilled water by gavage, using a 22 G intubation needle (Popper and Sons). Prior to DON treatment, mice received the same volume of distilled water using the same oral gavage procedure for a 7-day consecutive habituation period.

#### I.p. DON injection

For habituation, animals were handled and injected i.p. with physiological saline solution (0.9% NaCl) daily for at least 7 days prior to the experiment. DON was injected i.p. at a concentration of 1.25 mg/kg BW.

#### Fluorogold (FG) injection

To assess blood diffusion through CVOs, the dye FG (17514-AAT Hydroxystilbamidine, Euromedex, 30 µl at 2%, dissolved in 0.9% NaCl) was i.p. administered. Animals were killed 3 h after the FG injection.

### Food intake measurements

One hour before lights out, mice received either intra-esophageal or i.p. administration of DON or vehicle. Immediately after treatment, a pre-weighed fresh food was given. The measurement of food intake was the same as in previous studies [33]. Food intake was calculated as the difference between the pre-weighed and the remaining powder measured with a precision balance (0.01 g; Denver Instrument from Bioblock).

### Analysis of plasma samples

Retro-orbital blood samples were collected 40 min after *p.o.* administration of H_2_O or DON 1.25 mg/kg BW. Blood samples were centrifuged (3000×*g*, 15 min, 4 °C). Then, the plasmas were collected and quickly frozen at − 80 °C until use. The quantitative determination of plasma DON concentrations was assessed by enzymatic colorimetric method assay kit "Veratox High Sensitivity ELISA (Neogen, Scotland, UK) according to the manufacturer’s instructions with some modifications. Aliquots of DON standards (1–50 ng/ml) or diluted plasma samples (1:2 v/v) were mixed with diluted DON horseradish peroxidase conjugate [(1:7 v/v) in 1% (w/v) bovine serum albumin (sigma) in PBS] and then incubated in DON-antibody-coated microtiter wells for 45 min [34]. After incubation, wells were washed with H_2_O and K-Blue substrate was added and incubated for an additional 20 min. The reaction was stopped by stop solution and the plates were read at 650 nm on an ELISA microplate reader (Epoch2, BioTek Instruments, Vermont, USA). The DON concentrations in the samples were determined from the DON standard curve log (B/B_0_-B) = fct (log DON concentration).

### Immunohistochemistry procedures

The *p.o.*-treated animals used for immunostaining procedure were killed 3 or 6 h after treatment without free access to food. Mice were anaesthetized using i.p. injection of ketamine (120 mg/kg BW, Imalgène® 1000, Boehringer Ingelheim) and xylazine (16 mg/kg BW, Rompun® 2%, Bayer Santé). Intracardiac perfusion was achieved with 0.1 M PBS followed by 4% paraformaldehyde (PFA) in 0.1 M phosphate buffer. Brains were post-fixed for 1 h in 4% PFA at room temperature, rinsed in phosphate buffered saline (PBS) and then cryoprotected for 24–48 h in 30% sucrose at 4 °C. After freezing of the brains in isopentane (− 40 °C), coronal sections (40 µm thick) were cut on a cryostat (Leica CM3050, France) and serially collected in PBS (0.1 M; pH 7.4). Brains were cut from caudal brainstem (Bregma − 8.24 mm) to forebrain (Bregma + 0.75 mm). Immunohistochemistry conditions and antibodies used for labeling and cellular phenotyping are described in Additional file 6: Table S1. Non-specific binding was assessed on alternate slices which were treated identically but in which the primary antibody was omitted. The c-Fos immunochemistry was performed as previously described [35]. Finally, all sections were mounted on gelatin-coated slides, air dried, and coverslipped with mounting medium for fluorescence microscope preparation (DAKO) (Table 1).

**Table 1:** Immunohistochemistry conditions

Immunostaining	Primary antibody	Secondary antibody	Serum blocking
IBA-1	Goat polyclonal anti-IBA1 (1:500) ab5076, Abcam	Donkey anti-Goat Alexa Fluor 488 (1:400) A11055, Invitrogen	Horse serum 3%
c-Fos	Rabbit polyclonal anti-c-Fos (1:3000) ABE457, Merck Millipore	Biotinylated Goat anti-Rabbit (1:400) BA-1000, Vector Labs	Normal goat serum 3%
IBA-1/CD68	Rabbit polyclonal anti-IBA1 (1:4000) 019-19741, Fujifilm Wako Mouse monoclonal anti-CD68 (1:200) E-AB-22013, Elabscience	Donkey anti-Rabbit Alexa Fluor 594 (1:400) A21207, Invitrogen Goat anti-Mouse Alexa Fluor 488 (1:400) A11029, Invitrogen	Bovine serum albumine 3% Bovine serum albumine 3%
IBA-1/CD206	Rabbit polyclonal anti-IBA1 (1:4000) 019-19741, Fujifilm Wako Goat polyclonal anti-CD206 (1:500) AF2535, R&D Systems	Donkey anti-Rabbit Alexa Fluor 594 (1:400) A21207, Invitrogen Donkey anti-Goat Alexa Fluor 488 (1:400) (A11055, Invitrogen)	Bovine serum albumine 3% Bovine serum albumine 3%
IBA-1/CD11b	Rabbit polyclonal anti-IBA1 (1:4000) 019-19741, Fujifilm Wako Rat monoclonal anti-CD11b (1:100) Sc-23937, Santa Cruz Biotechnology	Donkey anti-Rabbit Alexa Fluor 488 (1:400) A21206, Invitrogen Goat anti-Rat Alexa Fluor 594 (1:400) A11007, Invitrogen	Bovine serum albumine 3% Bovine serum albumine 3%
CD11b/CD68	Rat monoclonal anti-CD11b (1:100) Sc-23937, Santa Cruz Biotechnology Mouse monoclonal anti-CD68 (1:200) E-AB-22013, Elabscience	Goat anti-Rat Alexa Fluor 594 (1:400) A11007, Invitrogen Goat anti-Mouse Alexa Fluor 488 (1:400) A11029, Invitrogen	Bovine serum albumine 3% Bovine serum albumine 3%
IBA-1/TMEM119	Goat polyclonal anti-IBA1 (1:500) ab5076, Abcam Rabbit polyclonal anti-TMEM119 (1:2000) GTX134087, GeneTex	Donkey anti-Goat Alexa Fluor 488 (1:400) A11055, Invitrogen Donkey anti-Rabbit Alexa Fluor 594 (1:400) A21207, Invitrogen	Horse serum 3% Bovine serum albumine 3%
TMEM119/CD68	Rabbit polyclonal anti-TMEM119 (1:2000) GTX134087, GeneTex Mouse monoclonal anti-CD68 (1:200) E-AB-22013, Elabscience	Donkey anti-Rabbit Alexa Fluor 594 (1:400) A21207, Invitrogen Goat anti-Mouse Alexa Fluor 488 (1:400) A11029, Invitrogen	Bovine serum albumine 3% Bovine serum albumine 3%
FG/CD68	Rabbit polyclonal anti-FG (1:1000) AB153, Chemicon Mouse monoclonal anti-CD68 (1:200) E-AB-22013, Elabscience	Donkey anti-Rabbit Alexa Fluor 594 (1:400) A21207, Invitrogen Goat anti-Mouse Alexa Fluor 488 (1:400) A11029, Invitrogen	Normal Goat serum 3% Bovine serum albumine 3%
FG/IBA-1	Rabbit polyclonal anti-FG (1:1000) AB153, Chemicon Goat polyclonal anti-IBA1 (1:500) ab5076, Abcam	Donkey anti-Rabbit Alexa Fluor 594 (1:400) A21207, Invitrogen Donkey anti-Goat Alexa Fluor 488 (1:400) (A11055, Invitrogen)	Normal Goat serum 3% Horse serum 3%

### Microscopy, image analysis and quantification

The c-Fos immunostaining was further analyzed by counting the positive nuclei on four distinct sections. Counting of c-Fos positive nuclei was performed on photomicrographs acquired using a tenfold lens with a DXM 1200 Camera (Nikon) coupled to ACT-1 software. The microscope was set at a specific light level, as was the camera exposure time. The c-Fos-positive nuclei were then counted on these pictures by computer-assisted morphometry using the NIH Image J software. Images were normalized by subtracting the background determined for each of the structures studied. The c-Fos-stained elements were identified by setting a threshold value (140 grey levels above the background on a 0–255 intensity scale). Counts were manually corrected for overlapping cell nuclei that were counted by the software as single. The software-generated counts of c-Fos-stained profiles were also manually corrected by excluding positive objects with an area no longer than 10 pixels (image resolution 150 pixels/in.) corresponding to objects with a surface equal or inferior to 3 square µm.

Fluorescent images were acquired on a confocal microscope (Zeiss LSM 710). In double-labeling experiments, images were acquired sequentially. To evaluate the ionized calcium-binding adaptor protein-1 (IBA1) labeling in the brainstem and hypothalamus, analysis was performed using the Image J software (NIH, USA). Briefly, images of coronal sections, at selected levels, were first captured using a tenfold lens with a DXM 1200 Camera (Nikon) coupled to ACT-1 software. The microscope was set at a specific illumination level, as was the camera exposure time digital camera. Additional file 1: Fig. S1 shows an overview of the method used to quantify IBA1 staining. The region of interest (ROI) was selected from row IBA1 image (Additional file 1: Fig. S1A, B). Since IBA1 staining yields higher staining intensity in the cell bodies than most of the dendritic processes, the brightness and contrast were adjusted to allow microglia processes visualization (Additional file 1: Fig. S1C). Using the threshold and size filter functions (Additional file 1: Fig. S1D, E), dark and small (< 14 µm^2^) particles were excluded from analysis. Thus, the number of IBA1 + cells per mm^2^, the mean surface of IBA1 + cells (µm^2^) were measured. Finally, the total IBA1 labeling area for a given ROI was calculated. We used high-power *z*-stack confocal imaging with IMARIS bitplane software (version 9.9.1) to identify and quantify internalized FG in CD68+ microglia as previously described [36, 37].

### Brainstem slice preparation and fluorescent latex beads uptake

The uptake of fluorescent amine-modified polystyrene latex beads (1 µm diameter, Merck) was assessed on acute brainstem slices prepared from 7- to 8-week-old mice fed ad libitum. Mice were deeply anesthetized (ketamine 120 mg/kg BW and xylazine 16 mg/kg BW in NaCl 0.9%, injected intraperitoneally) before decapitation. The brains were removed and placed in ice-cold oxygenated (95% O_2_–5% CO_2_) sucrose-enriched artificial cerebrospinal (aCSF) fluid containing in mM: 87 NaCl; 75 sucrose; 25 NaHCO_3_; 10 Glucose; 7 MgCl_2_, 2.5 KCl, 1 Na_2_HPO_4_; 0.5 CaCl_2_; osmotic concentration 320–330 mOsm. Hindbrains were embedded in agarose (1.5% agarose in sucrose-enriched aCSF) and coronal slices (250 µm thick) were cut with a compresstome (VF-200-0z, Precisionary). Slices selected to contain the AP were transferred to a constantly oxygenated holding chamber (95% O_2_–5% CO_2_) containing sucrose-enriched aCSF for 30 min, then 30 min in the recording aCSF used for the bead’s uptake assay containing in nM: 119 NaCl; 26.2 NaHCO_3_; 11 glucose; 2.5 KCl; 2.5 CaCl_2_; 1.3 MgCl_2_; 1 Na_2_HPO_4_; 320–330 mOsm. To assess phagocytic activity in microglia cells, slices were incubated in the presence of amine-modified polystyrene latex beads 1/1000 dilution, fluorescent yellow-green, mean particle size 1 μm; Sigma, UK) for 2 h. After two rinses in aCSF (15 min), slices were fixed in 4% PFA for 4 h at 4 °C. IBA1 labeling was performed as described above. Latex beads and IBA1 + co-localization were analyzed by confocal microscopy (Zeiss LSM 710).

### Statistical analysis

Data are represented as mean ± S.E.M. Comparisons between data from vehicle- and DON-treated mice were performed using unpaired 2-tailed Student’s *t*-test. One-way ANOVA was performed to compare data from mice treated with different doses of DON. Fisher’s LSD (least significant difference) test was used for post hoc analysis. *P* values less than 0.05 were considered significant. A 2-way repeated-measures ANOVA (*P* < 0.005) was performed in Figs. 3B, C, E and 12B, E followed by Bonferroni post hoc tests for individual time points.

## Results

### Microglial hypertrophy within CVOs in response to DON administration

**Fig. 1:**
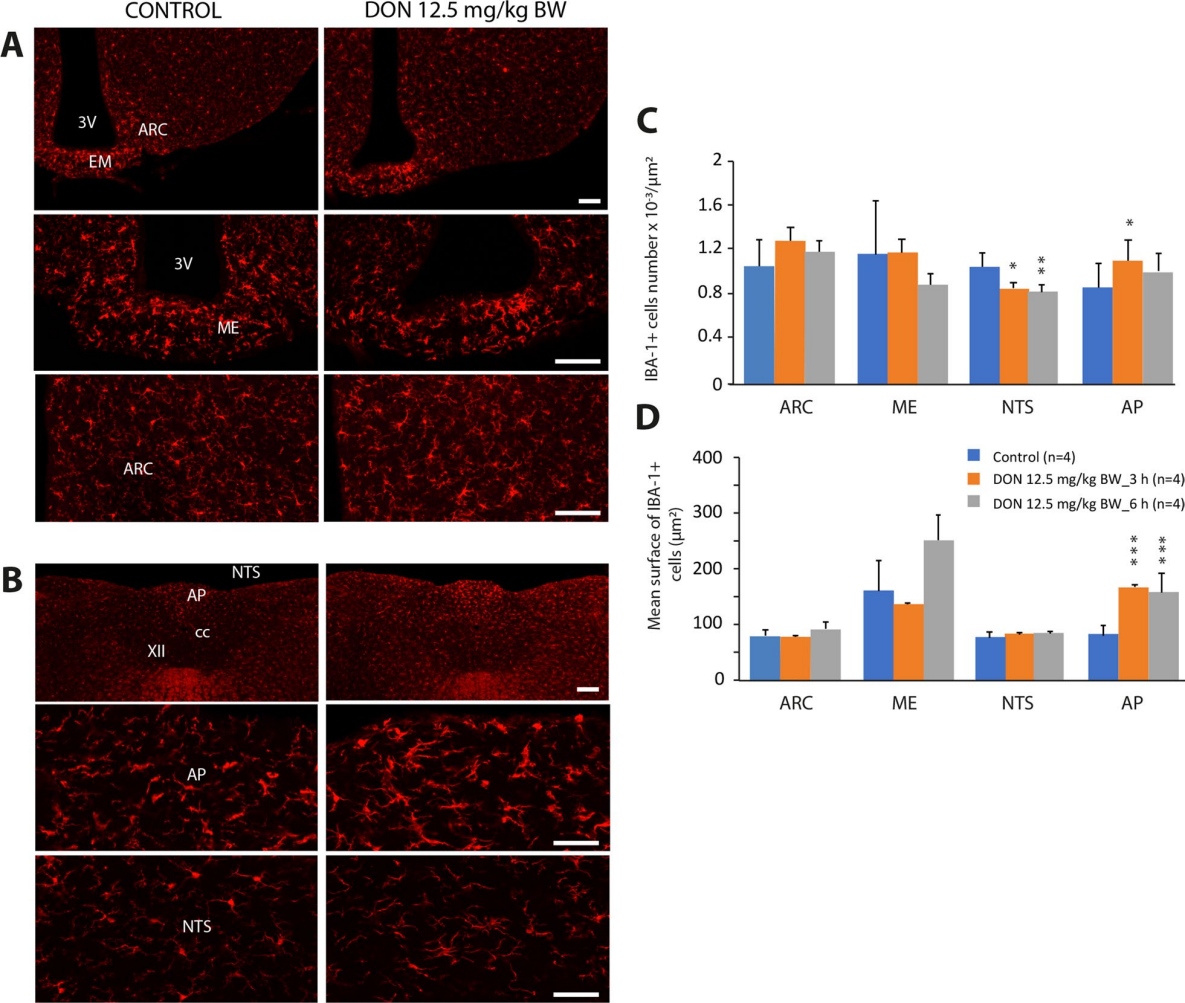
Effect of DON administration on IBA1 immunoreactivity within the hypothalamus and brainstem. **A**, **B** Representative images of IBA1-immunoreactivity in the hypothalamus (**A**) and brainstem (**B**) of control and DON-treated animals. Images originated from animals killed 6 h after treatment. High magnification images illustrate IBA1 immunoreactivity within two circumventricular organs, i.e., ME and AP and adjacent integrative nuclei ARC and NTS. Scale bars: 250 μm. **C**, **D** Quantitative analysis of IBA1 immunoreactivity performed at 3 and 6 h after treatment in selected regions of control and DON-treated animals. Histograms represented the number of IBA1 + cells per unit area (**C**) and the mean surface of IBA1 + cells (**D**). One-way ANOVA, *significantly different from respective control, *p < 0.05, **p < 0.01, ***p < 0.001. *AP* area postrema; *ARC* arcuate nucleus; *cc* central canal: *ME* median eminence; *XII* hypoglossal nucleus; *NTS* nucleus tractus solitarius. 3V: third ventricle

**Fig. 2:**
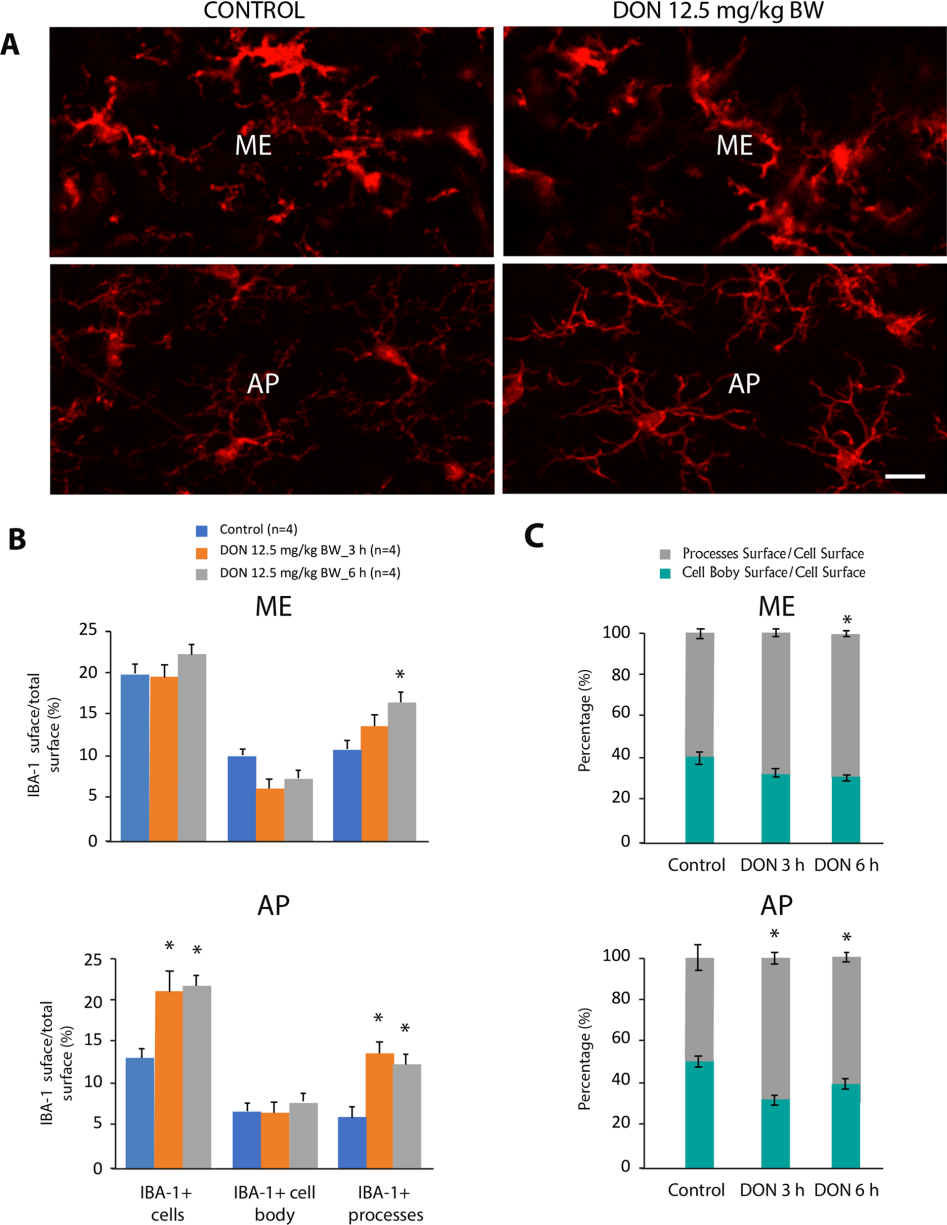
Effect of DON administration on IBA1 + cell morphology. **A** Representative images of IBA1-immunoreactivity in the ME (upper panels) and AP (lower panels) of control and DON-treated animals. Images originated from animals killed 6 h after treatment. Scale bar: 10 µm. **B** Quantitative analysis of IBA1 + cell surface, IBA1 + cell body area and IBA1 + process surface expressed as a percentage of total ME (upper panel) or AP (lower panel) area. **C** The cell body to cell process ratio of microglia in the ME (upper panel) and AP (lower panel) of control and DON-treated animals. One-way ANOVA, *p < 0.05. *AP* area postrema; *ME* median eminence

First, we sought to determine whether DON, administered at a dose known to induce central inflammation and anorexia, i.e., 12.5 mg/kg BW [31], modified microglial distribution and morphology in the hypothalamus and DVC. For this purpose, IBA1 immunolabeling was performed at 3 and 6 h after DON treatment of hypothalamic and brainstem sections. DON-induced anorexia was still present at both times points [31]. IBA1 staining was examined at a confocal microscopic level at the hypothalamus and DVC control and DON-treated animals (Fig. 1A, B). From these IBA1 immunostaining images, we first performed a quantification of the number of IBA1 + cells reported on the surface of the studied structures (Fig. 1C). We observed a slight decrease in the number of IBA1 + cells in the nucleus tractus solitarius (NTS) while this index slightly increased within the AP 3 h after DON treatment. When we looked at the average area of IBA1 cells, we only saw an increase in the AP either at 3 or 6 h after DON treatment (Fig. 1D). No difference was observed in the hypothalamus (Fig. 1C, D). Next, we carried out a finer analysis of microglial cell morphology in the ME and AP by quantifying the surface area of the processes versus the surface area of cell body (Fig. 2). This analysis showed an increase in the surface area of microglial processes at 6 h after DON administration in the ME and at both time points studied in the AP (Fig. 2B). This appears to reflect microglial hypertrophy in response to DON in the CVOs studied (Fig. 2C). It should be noted that in resting conditions, the morphology of microglial cells is significantly different between the AP and the median eminence (ME). Within the AP, microglial cells were branched with a small cell body, whereas in ME the cells had a more amoeboid shape with shorter processes (Fig. 2). We simultaneously immunostained glial fibrillary acidic protein (GFAP) to track any potential astrocyte reaction to DON. Compared to control animals at 3- and 6-h post-treatment, no difference in the GFAP reactivity was detectable in the animals treated with 12.5 mg/kg BW DON (Additional file 2: Fig. S2).

### Microglia deletion exacerbated the anorectic action of DON

**Fig. 3:**
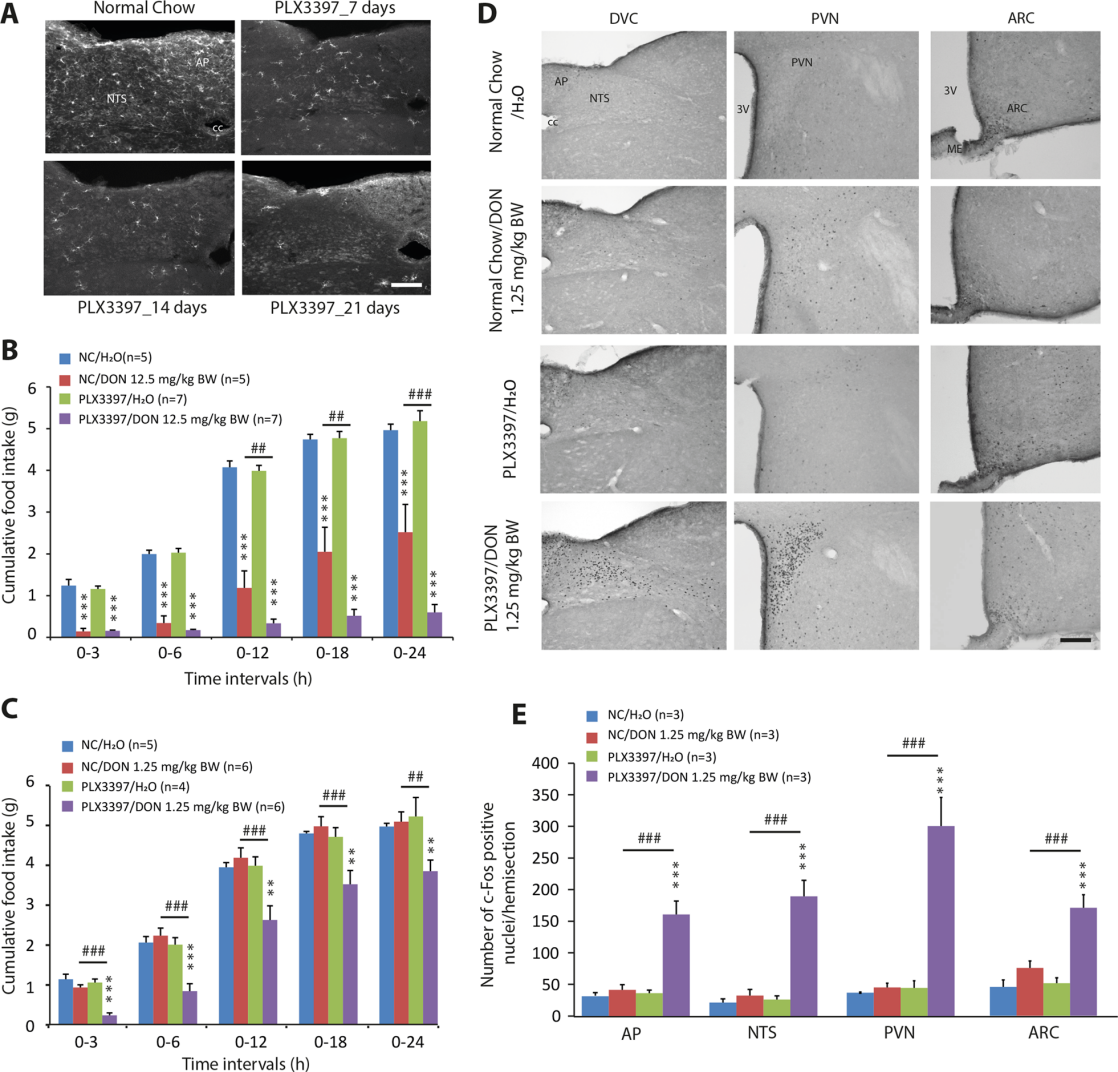
PLX3397-induced microglia deletion exacerbates DON-induced anorexia. **A** Representative images of IBA1 staining in the dorsal vagal complex (DVC) of animal fed for 3 weeks with normal chow (NC) or food supplemented with PLX3397 (290 mg/kg chow). Scale bar: 100 µm. **B–C** Cumulative food intake (g), measured over a 24-h period, of control or PLX3397-treated mice given *p.o.* administration of either vehicle (H_2_O) or DON 12.5 (**B**) or 1.25 (**C**) mg/kg BW. A 2-way repeated-measures ANOVA (*p* < 0.05) was performed in **B** and **C** followed by post hoc Bonferroni tests for individual time points. Differences in food intake between vehicle- and DON-treated groups was observed (**p < 0.01, ***p < 0.001). PLX3397 diet induced a significant difference in food intake of DON-treated groups (^##^*p* < 0.01, ^###^*p* < 0.001). **D.** Microphotographs illustrating c-Fos protein labeling observed with DVC (left), PVN (middle) and ARC (right) of control or PLX3397 mice treated with either vehicle or DON 1.25 mg/kg BW and killed 3 h after treatment. Scale bar: 200 µm. **E.** Quantification of c-Fos-positive cells in structures of interest. A 2-way repeated-measures ANOVA (*p* < 0.05) was performed followed by post hoc Bonferroni tests for individual time points. Differences in c-Fos expression between vehicle- and DON-treated groups was observed (***p < 0.001). The PLX3397 diet induced a significant difference between DON-treated groups (^##^*p* < 0.01, ^###^*p* < 0.001). *AP* area postrema; *ARC* arcuate nucleus; *cc* central canal; *DVC* dorsal vagal complex; *ME* median eminence; *NTS* nucleus tractus solitarius; *PVN* paraventricular nucleus; 3 V: third ventricle

Considering the increase in microglial reactivity in the AP and to a lesser extent in the ME, in response to DON administration, we then sought to evaluate anorectic DON’s action after microglia deletion. PLX3397 is an orally bioavailable selective CSF1R/c-kit inhibitor that crosses the blood brain barrier (BBB) and has been reported to reduce microglia survival in vivo [32, 38]. Based on these previous studies, animals were fed chow containing 290 mg/kg PLX3397 for 3 weeks. IBA1 immunostaining performed on brainstem and hypothalamic sections at 7, 14 and 21 days after PLX3397's introduction into the diet confirmed the progressive deletion of microglia in these regions (Fig. 3A). At 21 days, IBA1 expression was strongly reduced (~ 95% reduction) by PLX3397 treatment in the DVC (Fig. 3A) and hypothalamus (not shown). Note that PLX3397 treatment did not modify the food intake and body weight of treated mice when compared to normal chow-fed mice (data not shown). A single oral administration of DON at 12.5 mg/kg BW resulted in a decrease in cumulative food intake over 24 h in NC-and PLX3397-fed mice (Fig. 3B). Interestingly, DON-induced anorexia is statistically higher in PLX3397 animals than in control animals (Fig. 3B). DON reduced food intake by 38.5% and 86.5% in NC- and PLX3397-treated mice, respectively, during the first 24 h after administration. Furthermore, DON failed to lower food intake in control mice when given at 1.25 mg/kg BW, but did so significantly in the PLX3397 group (Fig. 3C). Over 24 h, the decrease in food intake induced by DON 1.25 mg/kg BW in PLX3397-treated mice was 18.25%.

### Microglia deletion enhanced the number of cells activated in response to DON administration

Previous studies have shown that *p.o.* administration of DON at 12.5 mg/kg BW causes specific activation of central structures such as the DVC and hypothalamic nuclei [24, 25, 31, 39]. We then quantified cellular activation in response to DON 1.25 mg/kg BW using immune detection of the early gene c-Fos. Animals were killed 3 h after DON administration, a time point where anorexia is ongoing. A very low basal level of c-Fos-positive nuclei was observed in the brainstem and hypothalamus of NC-fed animals treated with H_2_O or DON 1.25 mg/kg BW (Fig. 3D). Similarly, PLX3397-treated mice that received water exhibited a low level of c-Fos + cells (Fig. 3D). On the other hand, DON 1.25 mg/kg BW induced strong c-Fos labeling in the DVC and hypothalamus of PLX3397-treated animals (Fig. 3D). Quantification of c-Fos + cells confirmed the strong stimulation induced by DON 1.25 mg/kg BW in the AP, NTS, arcuate nucleus (ARC), and paraventricular nucleus (PVN) of PLX3397-treated mice (Fig. 3E).

### DON hypersensitivity in PLX3397-induced did not result from an increased intestinal absorption

Since PLX3397 is an additive in animal feed and DON is administered by gavage, we wanted to see if a disruption of the intestinal barrier by DON [40] could lead to increase its absorption and explain the observed hypersensitivity. Plasmatic doses demonstrated that both NC- and PLX3397-treated mice receiving *p.o.* DON experienced a rise in blood DON levels. No significant difference was observed between the control and PLX3397 groups, which received DON at a concentration of 1.25 mg/kg BW (Additional file 3: Fig. S3A). To by-pass the intestinal absorption of DON, we next performed an i.p. injection of DON and measured food intake over a 6-h period. After its i.p. injection, DON 1.25 mg/kg BW induced a reduction in food intake similar to that observed after its administration by gavage (Additional file 3: Fig. S3B). *P.o.* and i.p. DON administration reduced food intake by 77.7 and 61.9% after 3 h, respectively. Similarly, the reduction of food intake was 62.5 and 53.3% 6 h after its oral or i.p. administration, respectively (Additional file 3: Fig. S3B). The same DON dose, 1.25 mg/kg BW, had no effect on food intake in control animals regardless of the route of administration (Additional file 3: Fig. S3C and data not shown).

### Multiple microglial phenotypes coexist within the DVC and hypothalamus and are modified by DON treatment

**Fig. 4:**
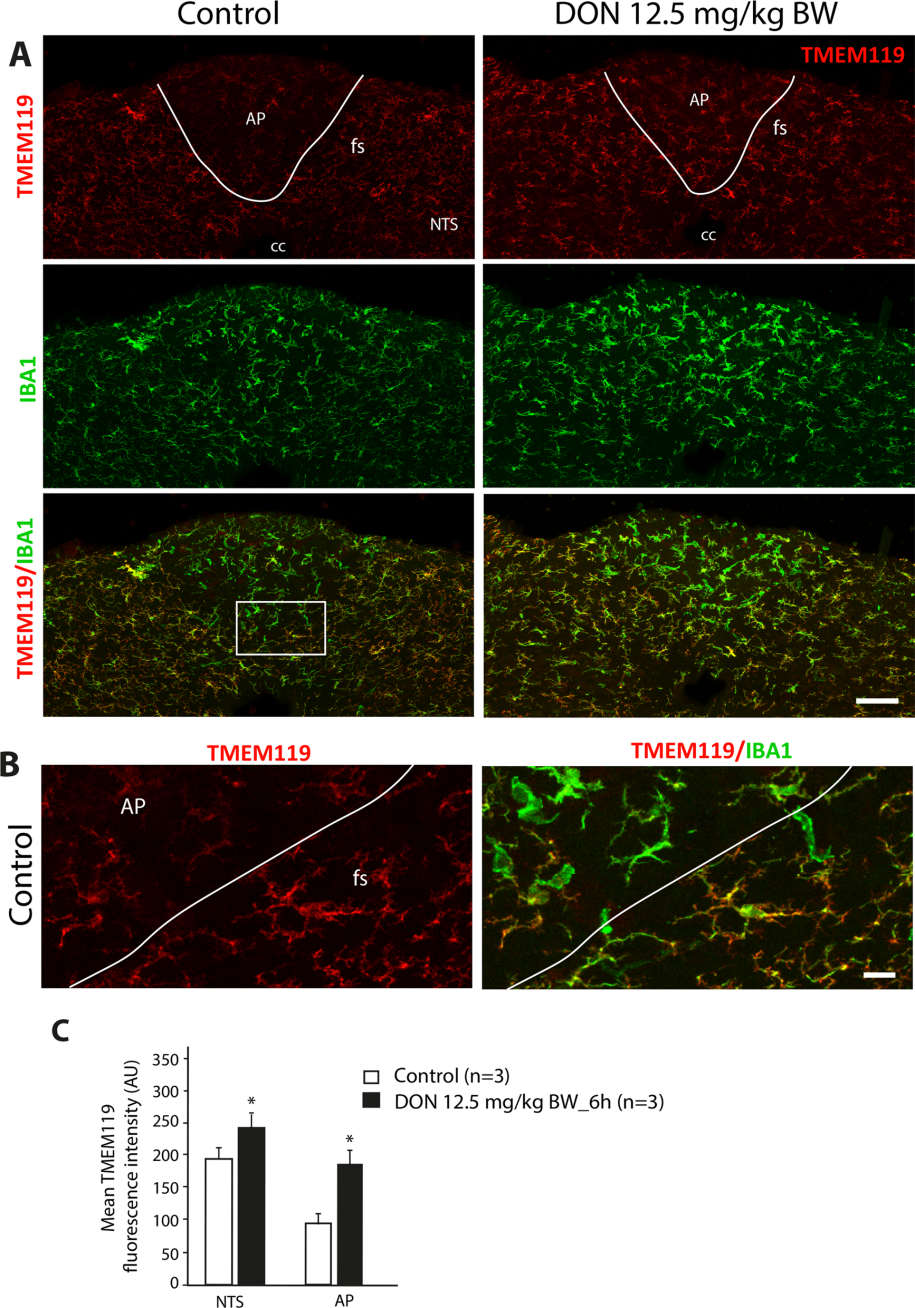
TMEM119 expression within the DVC of control and DON-treated animals. **A** Representative photomicrographs of TMEM119 and IBA1 labeling taken from coronal brainstem sections of mice treated with vehicle or DON. The white lines symbolize the border between AP and NTS. **B** Representative images of double TMEM119/IBA1 labeling highlighting the phenotype of microglial cells located within the AP and the *funiculus separans* of control mice*.* The white box indicated regions from panels **A** where panel **B** originated. **C** Quantification of TMEM119 labeling expressed in mean gray level in subregions of the DVC of control and DON-treated animals (12.5 mg/kg BW; *p < 0.05). Scale bars: 500 µm in **A**, 10 µm in **B**. *AP* area postrema; *cc* central canal; *fs* *funiculus separans*; *NTS* nucleus tractus solitarius

**Fig. 5:**
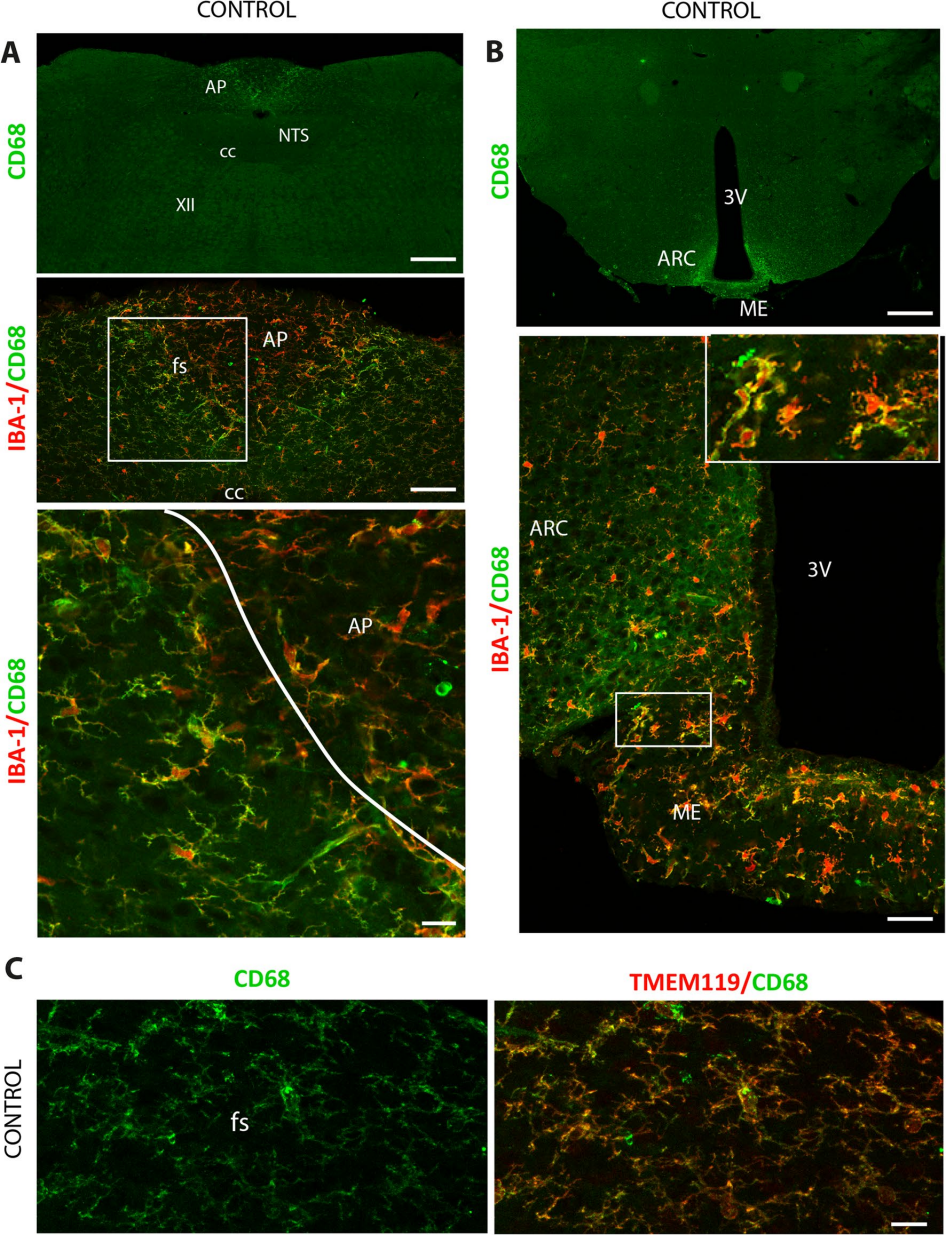
CD68+ microglia are present within the DVC and hypothalamus of control animals. **A** Representative photomicrographs of CD68 labeling performed on brainstem coronal sections of control mice. Note the localized expression of CD68 within the *funiculus separans* and postremal NTS. *Lower panels* High magnification images of double IBA1/CD68 labeling highlighting the presence of different microglial cell phenotypes within the AP, *funiculus separans* and NTS*.* The white box indicated regions where inset originated*.* **B** Representative photomicrographs of CD68 labeling performed on hypothalamic coronal sections of control mice. Note the localized expression of CD68 within the ARC/ME border. *Lower panel* High magnification images of IBA1/CD68 double-labeling highlighting the presence of different microglial cell phenotypes within the ARC and ME*.* The white box at **A** indicated the regions where inset originated. **C** High magnification images of double CD68/TMEM119 labeling performed at the *funiculus separans* level. Scale bars: 200 µm in **A**–**B**, 50 µm in **C**. *ARC* arcuate nucleus; *AP* area postrema; *cc* central canal; *fs* *funiculus separans*; *ME* median eminence; *NTS* nucleus tractus solitaries; 3 V, third ventricle; XII: hypoglossal nucleus

**Fig. 6:**
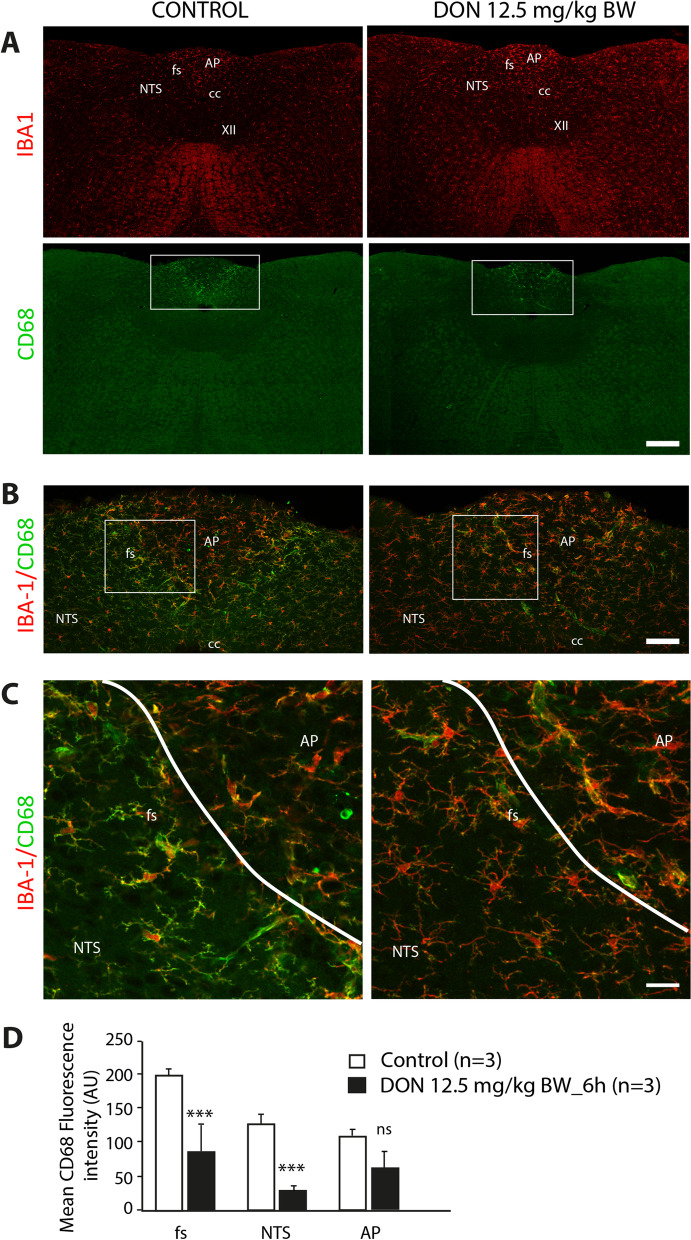
Effect of DON administration on CD68 microglial expression. **A** Representative photomicrographs of IBA1 and CD68 labeling taken from coronal brainstem sections of mice treated with vehicle or DON. Scale bar: 500 µm. **B** Representative images of double IBA1/CD68 labeling highlighting the phenotype of microglial cells located in the AP, *funiculus separans* and NTS of vehicle or DON-treated mice*.* The white boxes indicated regions from panels **A** which panels **B** originated. Scale bar: 50 µm. **C** High magnification photographs of the regions indicated by the box in panel **B,** respectively, illustrating the change in microglial phenotype in response to DON treatment particularly in the *funiculus separans*. The white lines symbolize the border between the AP and the NTS. Scale bar: 10 µm. *AP* area postrema; *cc* central canal; *fs* *funiculus separans*; *NTS* nucleus tractus solitarius; *XII* hypoglossal nucleus. **D** Quantification of CD68 labeling expressed in mean gray level in DVC subregions of control and DON-treated animals (12.5 mg/kg BW; ***p < 0.001, *ns* non-significantly different).

**Fig. 7:**
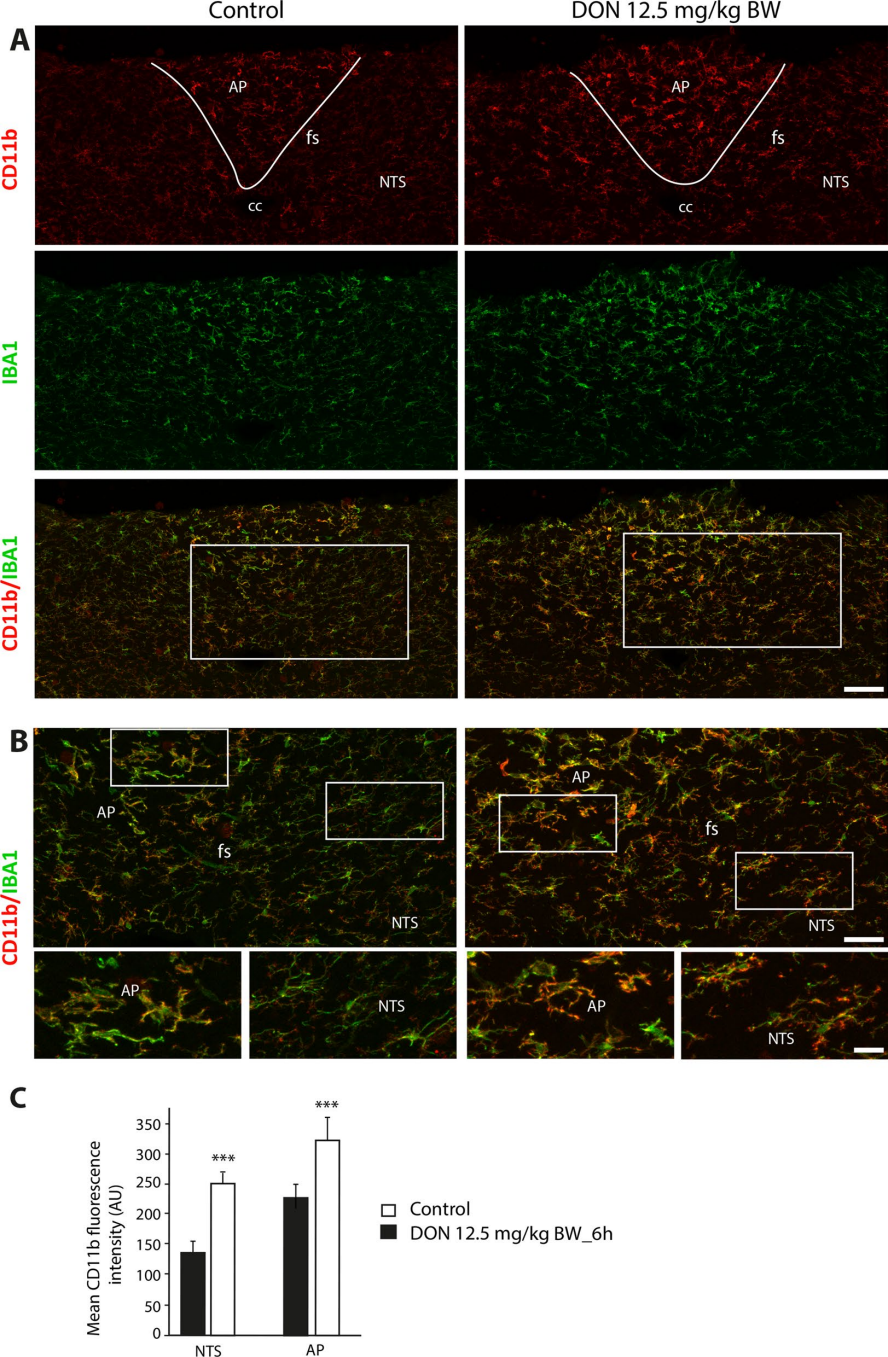
CD11b + expression within the DVC of control and DON-treated animals. **A** Representative photomicrographs of CD11b and IBA1 labeling taken from coronal brainstem sections of mice treated with vehicle or DON. The white lines symbolize the border between the AP and the NTS. **B** Overlay photographs of CD11b and IBA1 labeling from coronal brainstem sections of mice treated with vehicle or DON. The white boxes in panels **A** indicated regions from which panels **B **originated. Insets illustrate the modification of microglial phenotype in response to DON treatment. Scale bars: 500 µm in A; 50 µm in B. AP: area postrema; cc: central canal; fs: *funiculus separans*; NTS: nucleus tractus solitarius. **C**. Quantification of CD11b labeling expressed in mean gray level in DVC subregions of control and DON-treated animals (12.5 mg/kg BW; ***p < 0.001)

**Fig. 8:**
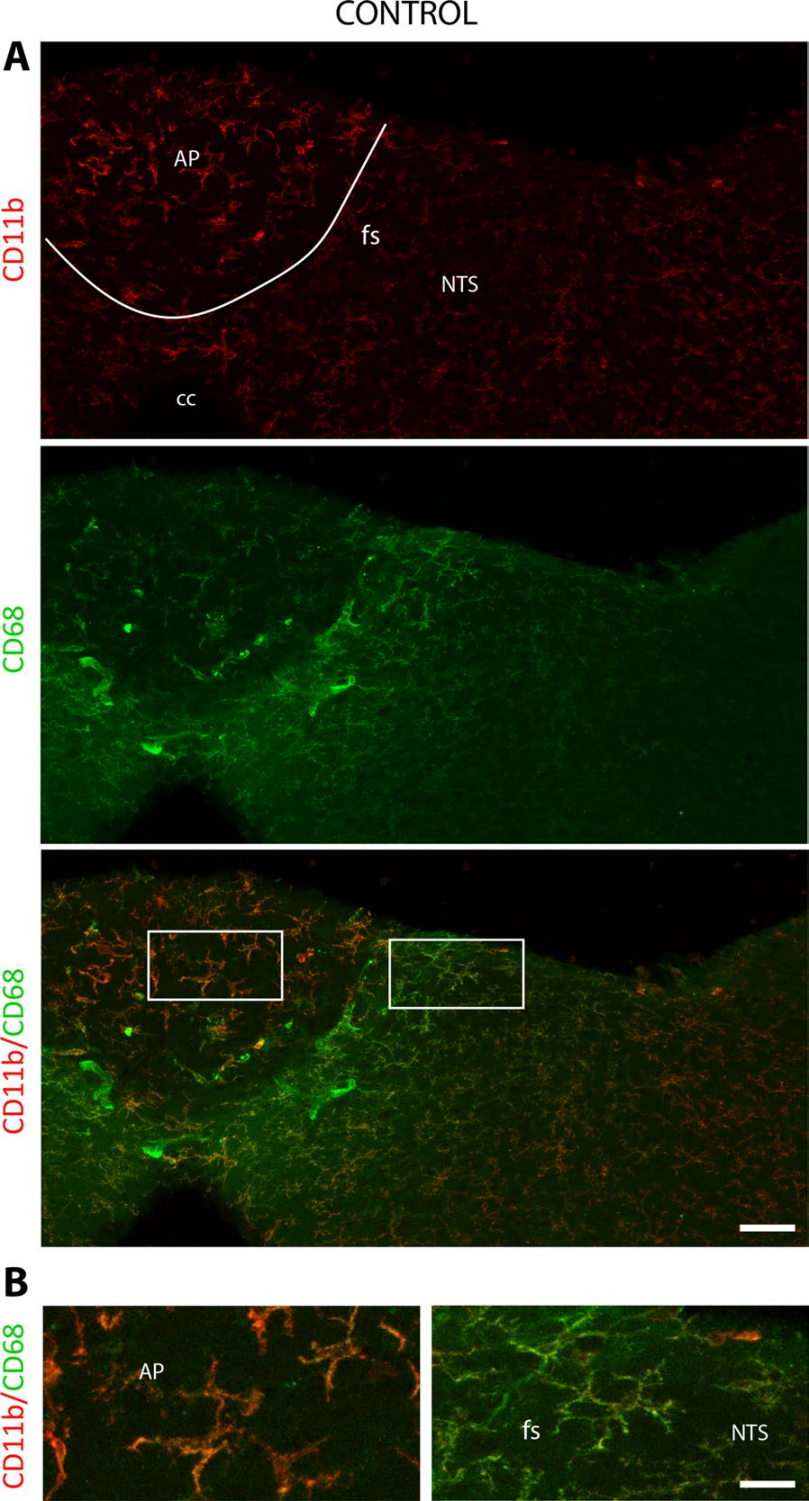
CD68 and CD11b expression reveal two distinct microglial populations within the DVC of resting animals. **A** Representative photomicrographs of CD11b and CD68 labeling taken from coronal brainstem sections of resting mice. The white line symbolizes the border between the AP and the NTS. The white boxes indicated regions from panel **A** where panel **B** originated. **B** Insets highlighted the differential expression of CD11b and CD68 of microglial cells located within the AP and *funiculus separans*/NTS. Scale bars: 500 µm in **A**; 10 µm in **B**. *AP* area postrema; *cc* central canal; *fs* *funiculus separans*; *NTS* nucleus tractus solitarius

We then investigated the phenotype and polarization of microglial cells located within the DVC and the hypothalamus using markers characteristic of pro-inflammatory or anti-inflammatory microglia, i.e., transmembrane protein 119 (TMEM119), cluster of differentiation receptors 11b (CD11b), CD68, and CD206. To determine whether DON, which induces microglial hypertrophy in the AP and the ME, also modified the phenotype of microglial subpopulations, the immunohistochemistry of previously mentioned markers was also performed on animals killed 6 h after receiving DON (12.5 mg/kg BW). TMEM119 labeling performed on the DVC and hypothalamus displayed a uniform pattern throughout the NTS (Fig. 4A) and ARC (data not shown). TMEM119 co-localized strongly with IBA1, confirming the microglial identity of the IBA + cells in these regions (Fig. 4A, B). In contrast, IBA + microglial cells located in the AP (Fig. 4A, B) and ME (data not shown) appeared less intensively stained with TMEM119. This revealed the existence of microglial populations with a different phenotype within the DVC and the hypothalamus (Fig. 4B). DON treatment (12.5 mg/kg BW) significantly increased TMEM119 expression within the AP and NTS (Fig. 4C). CD68 labeling revealed intense and narrow labeling at the AP/NTS border, i.e., the *funiculus separans (*Fig. 5A). This signal gradually faded in the lateral parts of the NTS. As for the AP, the labeling was very weak. Interestingly, although the CD68 and IBA1 markers are very strongly co-localized in the NTS and the AP, the expression levels of these two markers reveal different microglial phenotypes. Within the AP, cells strongly expressed IBA1 and weakly CD68 (IBA1+++/CD68+). In *funiculus separans*, a microglial subpopulation surrounding the AP exhibited an inverted phenotype with a strong CD68 expression and a weaker IBA1 signal (IBA1+/CD68+++). Finally, in the lateral parts of the NTS, microglia slightly expressed both IBA1 and CD68 (IBA1+/CD68+; Fig. 5A). A similar picture was found in the hypothalamus where CD68 was highly expressed by IBA1 + cells located at the interface between the ARC and the ME (Fig. 5B). CD68+ cells located in the *funiculus separans* were also found immunoreactive for TMEM119 (Fig. 5C). Interestingly, we observed that DON strongly reduced CD68 expression in the DVC (Fig. 6) and in the hypothalamus (data not shown). Microglia cells exhibiting IBA1 hyperactivity in response to DON within the AP and ME did not show increased expression of CD68 (Fig. 6A–C). Regarding CD11b labeling, microscopic analysis at the DVC and hypothalamic levels revealed that its expression was variable, showing robust expression in AP- and ME-restricted microglial cells while showing a lesser expression in the microglial cells of the NTS (Fig. 7) and ARC (data not shown). Treatment with DON 12.5 mg/kg BW increased CD11b expression in microglia located both in the AP and NTS (Fig. 7). Within the hypothalamus, DON treatment induced a similar modulation of CD11b expression (data not shown). Of note, CD68 and CD11b markers only partially co-localized at the DVC (Fig. 8) and hypothalamus (data not shown) levels, highlighting the variety of microglial phenotypes in these regions. In the *funiculus separans*, CD68-highly expressing microglia were weakly positive for CD11b (Fig. 8A, B). Conversely, CD11b-positive microglia that were confined within the AP weakly expressed CD68 (Fig. 8A, B). Regarding CD206, the labeling was mainly found in the AP, where it was distributed uniformly throughout the structure and often found associated with blood vessels (Additional file 4: Fig. S4). Within the NTS and hypothalamus, the CD206 signal was more diffuse and restricted to some blood vessels associated cells (Additional file 4: Fig. S4). Most of these CD206 positive cells can reasonably be assumed to be perivascular macrophages since they express neither IBA1 nor TMEM119 but are positive for CD206 and/or CD68 (Additional file 4: Fig. S4B and Fig. 8B). However, CD206/IBA1 double-immunolabeling revealed that a small proportion of microglia restricted to the AP and ME co-expressed both markers (5% of IBA1 + microglia co-expressed CD206; Additional file 4: Fig. S4 and data not shown). Finally, CD206 expression was not modified by DON treatment in either AP (Additional file 4: Fig. S4) or ME (data not shown).

### Microglial FG uptake within the brainstem and hypothalamus of resting animals

**Fig. 9:**
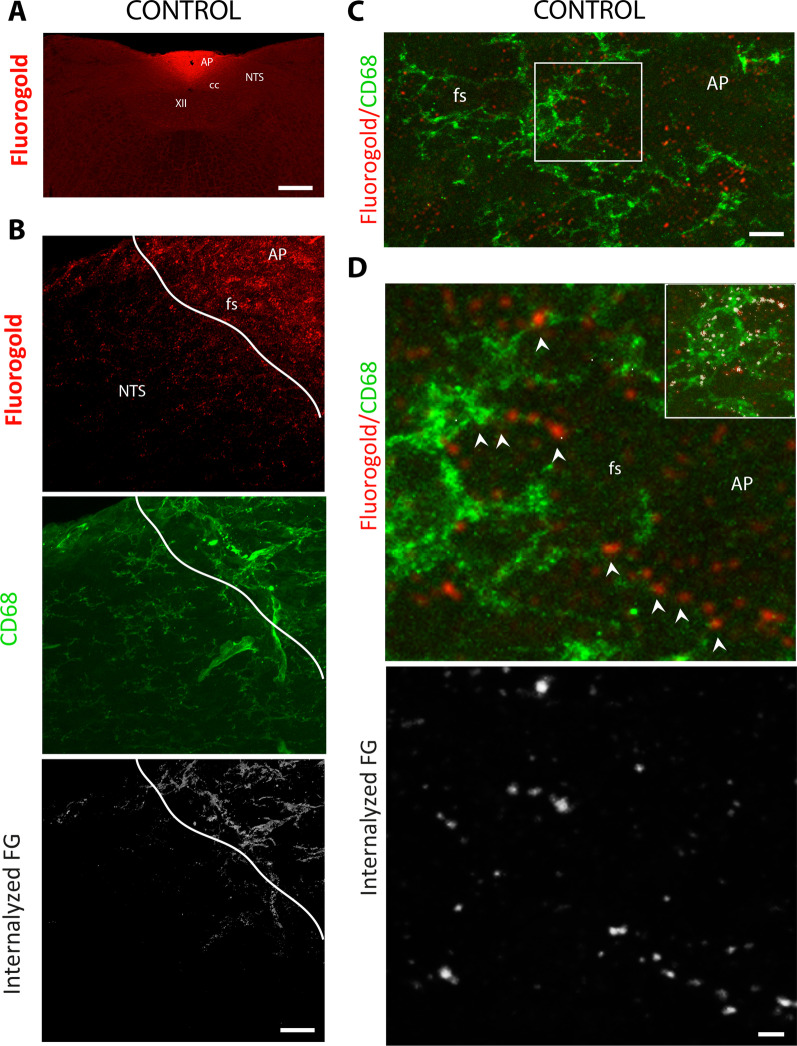
Brainstem CD68+ microglial cells exhibit endocytic activity at resting conditions. **A** A low-magnification photomicrograph of FG fluorescence observed at the brainstem. The animals were killed 3 h after the i.p. FG injection. Note the intense FG signal within the AP illustrating its diffusion from fenestrated capillaries. Scale bar: 500 µm. **B** Photomicrographs illustrating FG diffusion from surrounding AP structures where CD68+ microglia are present. *Lower panel* IMARIS bitplane software rendering of confocal Z-stacks was then used to visualize internalized FG within CD68+ microglia. Scale bar: 50 µm. **C** A representative high magnification confocal photomicrograph of FG signal and CD68+ immunostaining on DVC coronal section. Note the vicinity between CD68+ microglia and the punctiform FG signal. Scale bar: 20 µm. **D** Magnified photographs of the regions indicated by the box in panel C showing the co-localization of FG dot signal within CD68+ processes of microglia located in the *funiculus separans*. Arrowheads indicate the presence of FG punctiform signals enclosed in CD68+ processes. *Inset* IMARIS bitplane images of fluorogold and CD68 co-localization allowing visualization of internalized FG. *Lower panel* IMARIS bitplane image of co-localization allowing visualization of internalized FG in CD68+ microglia. Scale bar: 5 µm. AP: area postrema; cc: central canal; fs: *funiculus separans*; NTS: nucleus tractus solitaries; XII: hypoglossal nucleus

FG is a lipophilic molecule thought to cross cell membranes, entering the cell by endocytosis or phagocytosis. FG is widely used as a retrograde marker of axonal transport [41, 42]. FG does not cross the BBB and, after its i.p. injection, it does not label areas of the CNS protected by the BBB [43, 44]. Therefore, this route of administration has previously been used to label the AP [45, 46], thanks to the more permeable BBB characteristic of the CVO. In the present study, FG was administered by i.p. and animals were killed 3 h after injection. After i.p. FG injection, intense and diffuse FG staining was observed at low magnification in the AP while the NTS was mostly devoid of signal (Fig. 9A). Similarly, in the hypothalamus, a strong FG signal was observed within the ME while the ARC was weakly stained (Additional file 5: Fig. S5A). Higher magnification showed that the signal gradually faded from the AP but the *funiculus separans* and, to a lesser extent, the subpostremal NTS appeared labeled (Fig. 9B). Interestingly, this FG labeling gradient from AP to NTS was reminiscent of that already observed with the microglia marker, i.e., CD68 (Fig. 9B, C). The same observation was made at the hypothalamic level (Additional file 5: Fig. S5A). We then sought to determine whether the CD68+ microglial cells present in these areas had incorporated FG. Co-localization of FG and CD68+ was evaluated with IMARIS bitplane software. Figure 9C shows the co-localization of FG and CD68, which suggests that a portion of FG has been internalized. High confocal magnification of FG/CD68 dual labeling showed that CD68+ microglia located in the *funiculus separans* appeared positive for FG (Fig. 9D). Moreover, in these cells, the FG labeling is dot-shaped, suggesting of endocytosis/phagocytosis vesicles (Fig. 9D). These FG inclusions were clearly visible in CD68+ microglial processes (arrowheads in Fig. 9D). Internalization of FG in CD68+ cells was confirmed by IMARIS analysis (Fig. 9D). Quantification of internalized FG in CD68+ microglial revealed that 14.33 ± 3.52% of FG signal co-localized with CD68 labeling in the NTS. At the hypothalamic level, FG and CD68 co-localization performed through IMARIS analysis revealed the internalization of FG in CD68+ microglia of the ME and ARC (Additional file 5: Fig. S5B–D).

### Fluorescent latex bead uptake in brainstem slices of resting and DON-treated animals

**Fig. 10:**
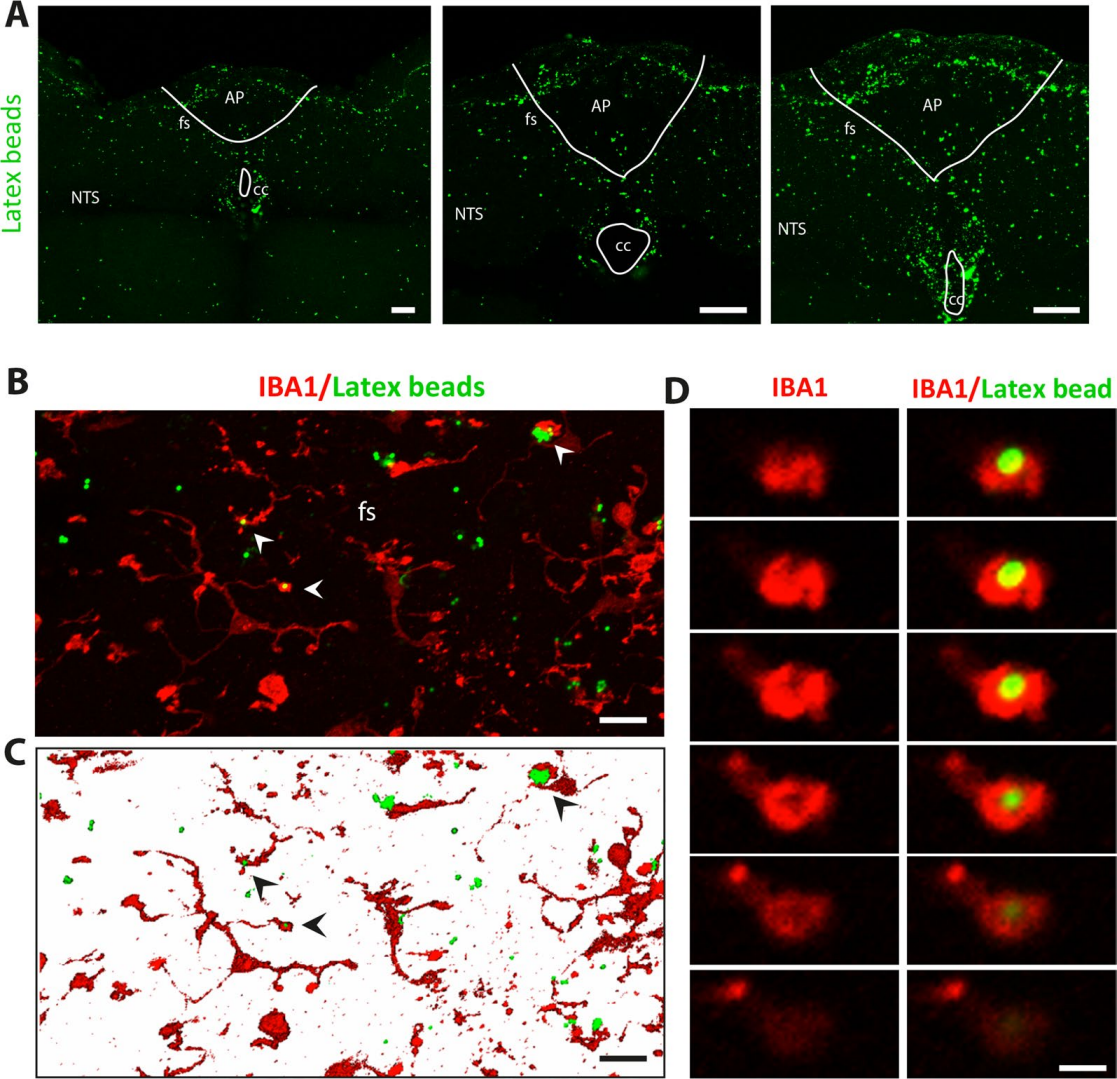
Brainstem microglia exhibit phagocytotic activity at resting conditions. **A** Representative confocal photographs of DVC slices surface showing a typical distribution of fluorescent latex beads mostly lodged in the AP, the *funiculus separans* and the subpostremal NTS. Scale bars: 200 µm. **B–C** Maximal xyz projection (**B**) and 3D reconstruction (**C**) of confocal images of IBA1 immunostaining and latex bead fluorescence acquired in the *funiculus separans* area. Arrowheads indicate latex beads engulfed in IBA1+ processes. Scale bar: 10 µm. **D.** Serial Z-stacking confocal microscope images of latex bead phagocytosis (green dots) by IBA1 + microglia. Scale bar: 3 µm. *AP* area postrema*;* *cc* central canal; *fs* *funiculus separans*; *NTS* nucleus tractus solitarius

**Fig. 11:**
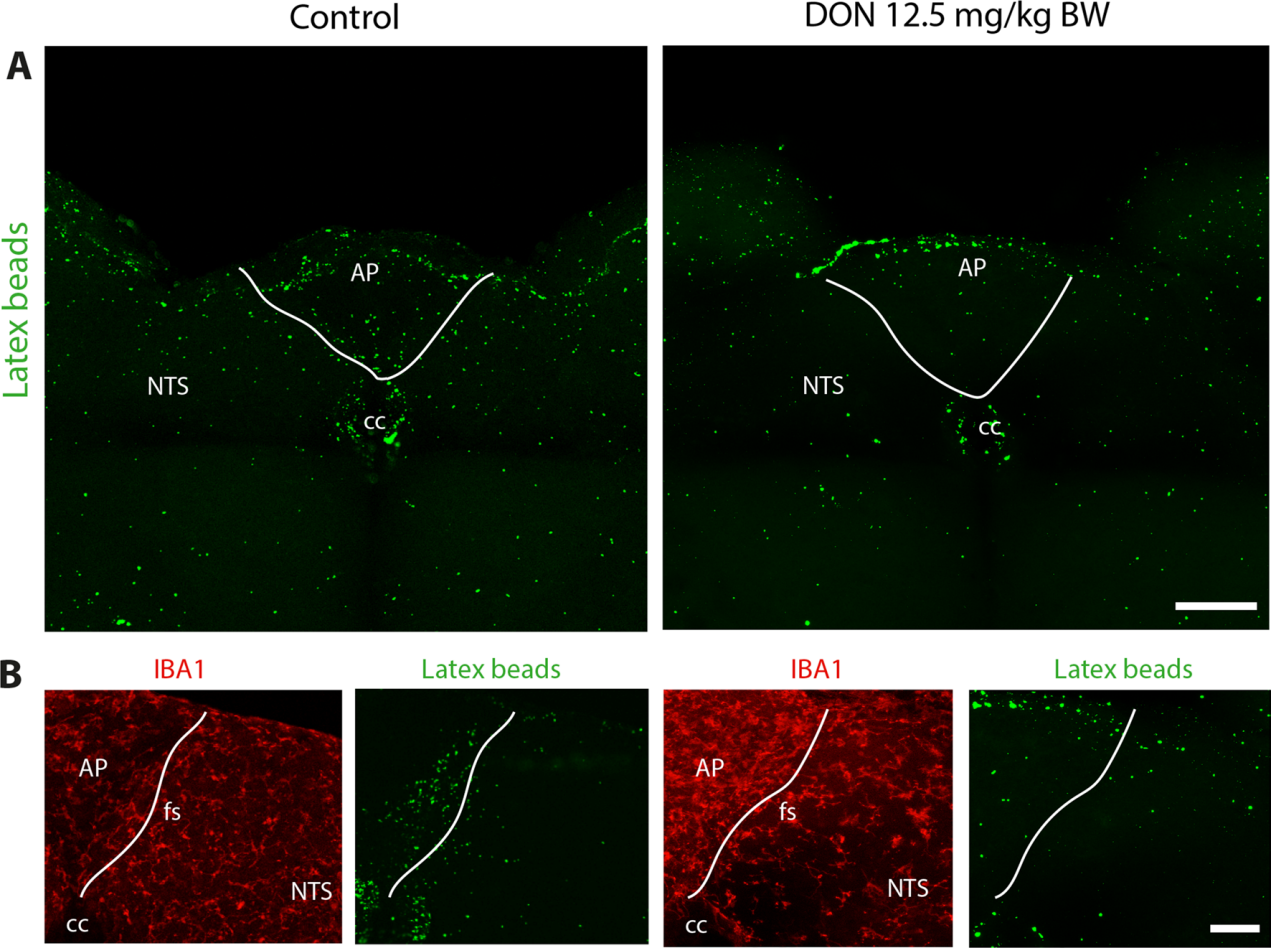
DON treatment reduced phagocytotic activity of brainstem microglia. **A** Representative confocal photographs of DVC slices surface showing a typical distribution of fluorescent latex beads of control and DON-treated (12.5 mg/kg BW, 6 h) animals. Scale bar: 200 µm**. B** Maximal xyz projection of confocal images of IBA1 immunostaining and latex bead fluorescence acquired in the *funiculus separans* area of control and DON-treated animals. Scale bar: 50 µm. *AP* area postrema*;* *cc* central canal; *fs* *funiculus separans*; *NTS* nucleus tractus solitarius

We then assessed phagocytic activity in brainstem microglia using fluorescent latex bead uptake. To achieve this goal, brainstem slices (250 µm) were incubated for 2 h with a solution containing fluorescent latex beads of 1 µm diameter. After 30-min recovery in aCSF without latex beads, slices were fixed and microglia stained with IBA1. Fluorescent latex beads were not homogenously distributed throughout the brainstem section, but an obvious higher density of latex beads was observable in and around the AP compared to other brainstem structures (Fig. 10A–C). Identification of microglial cells with IBA1 staining showed that these cells located in the AP and the *funiculus separans* were able to incorporate latex beads (Fig. 10D, E). Confocal z-stack images of IBA1/latex beads fluorescence allowed us to observe clear figures of latex beads phagocytosis by IBA1 + processes (Fig. 10F). Next, latex beads incorporation assay was performed using slices prepared from animals treated since 6 h with DON 12.5 mg/kg BW. Interestingly, DON treatment, which we have shown to reduce CD68 expression in microglial cells located within the funiculus separans, also decreased latex beads incorporation into slices at this AP/*funiculus separans* level (Fig. 11).

### Inhibition of constitutively active microglia exacerbated the anorectic action of DON

**Fig. 12:**
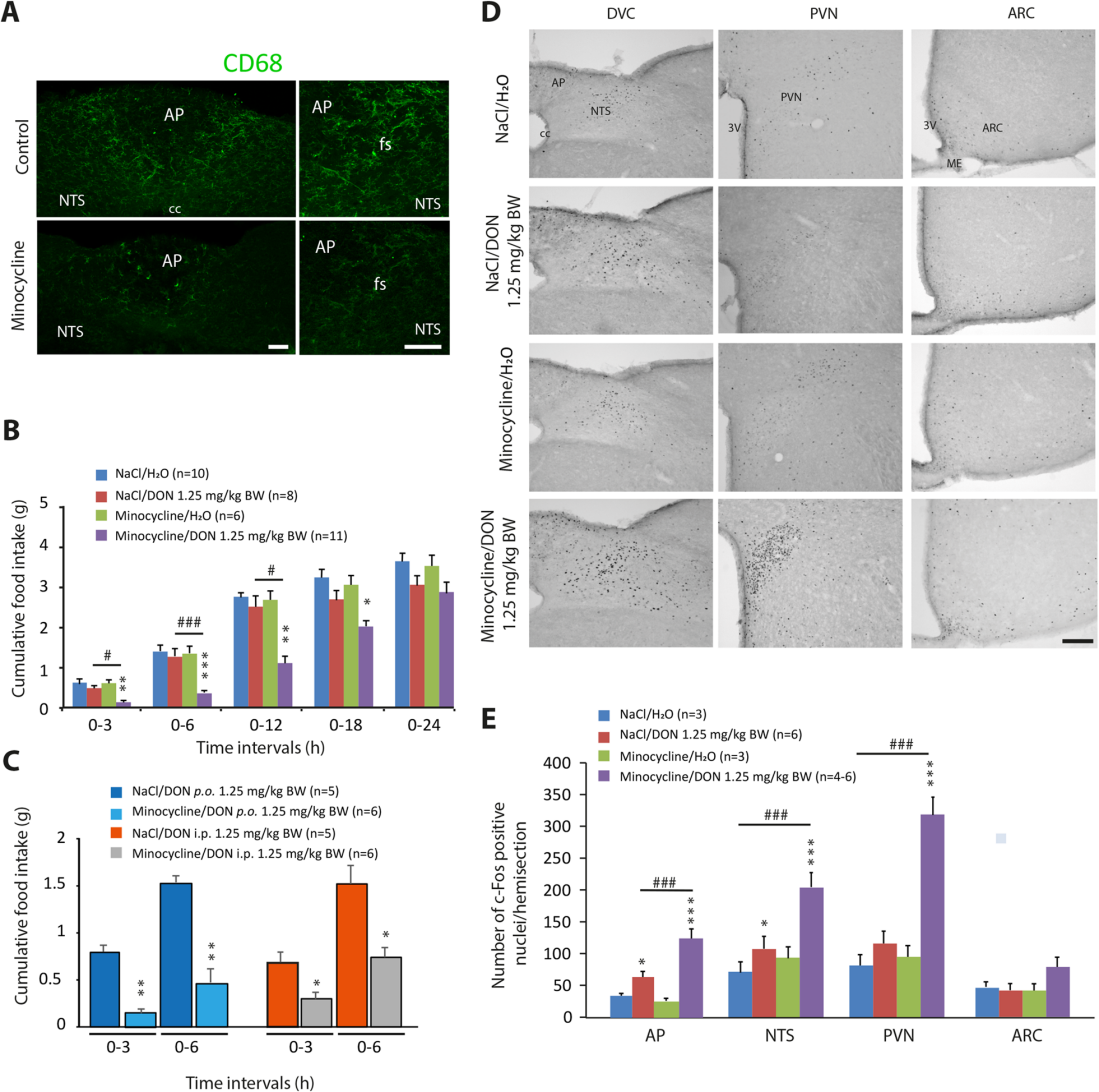
Microglia inhibition by minocycline exacerbates DON-induced anorexia. **A** Representative images of CD68 staining in the DVC of animals treated with vehicle or minocycline. Scale bars: 200 µm. **B** Cumulative food intake (g) of control or minocycline-treated mice after receiving *p.o.* administration of vehicle (H_2_O) or DON 1.25 mg/kg BW for 24 h. **C.** Cumulative food intake (g), measured over a 6 h period, of control or minocycline-treated mice given having received *p.o.* or *i.p.* administration of DON 1.25 mg/kg BW. A 2-way repeated-measures ANOVA (*p* < 0.05) was performed followed by Bonferroni post hoc tests for individual time points. *Significantly different from respective control group, *p < 0.05, **p < 0.01, ***p < 0.001. ^#^Significant difference between control and PLX3397 groups treated with DON; ^#^p < 0.05; ^###^p < 0.001. **D** Microphotographs illustrating c-Fos protein labeling observed in DVC (left), PVN (middle) and ARC (right) of control or minocycline-treated mice that received either vehicle or DON 1.25 mg/kg BW and were killed 3 h after treatment. Scale bar: 200 µm. **E** Quantification of c-Fos positive cells in structures of interest for control and treated with DON mice. A 2-way repeated-measures ANOVA (*P* < 0.05) was performed followed by Bonferroni post hoc tests for individual time points. Differences in c-Fos expression between vehicle- and DON-treated groups were observed (*p < 0.05; ***p < 0.001). PLX3397 diet induced a significant difference between DON-treated groups (^###^*p* < 0.001). *AP* area postrema; *ARC* arcuate nucleus; *cc* central canal; *DVC* dorsal vagal dorsal; *fs* *funiculus separans*; *ME* median eminence; *NTS* nucleus tractus solitaries; *PVN* paraventricular nucleus; 3 V: third ventricle

Minocycline is an antibiotic widely used as an inhibitor of microglial activation and polarization. Minocycline pretreatment has been reported to attenuate increased protein levels of IBA1 and other markers related to microglia activation [47–49]. Here, animals were injected with minocycline daily for three consecutive days before receiving DON 1.25 mg/kg BW or vehicle. Interestingly, minocycline treatment reduced CD68 reactivity in the AP and NTS of control animals (Fig. 12A). Oral administration of DON at 1.25 mg/kg BW resulted in a decrease in cumulative food intake over 24 h in minocycline-treated mice, while this dose remained ineffective in controls (Fig. 12B). The increased sensitivity to the anorectic action of DON appeared as early as 3 h post-treatment in minocycline-treated mice and lasted until 18 h after DON administration (Fig. 12B). As previously observed with PLX3397 treatment, the increased sensitivity to the anorectic action of DON was also observed in minocycline-treated animals receiving DON by i.p. injection (Fig. 12C). The immunostaining for c-Fos in response to DON 1.25 mg/kg BW showed an increased number of positive cells in the AP, NTS, and PVN of minocycline-pretreated mice (Fig. 12D, E). Note that no c-Fos labeling was observed in the ARC whatever the group considered (Fig. 12D, E).

## Discussion

The central mechanisms underlying the modulation of food intake by DON are not totally identified [21]. DON has been reported to modulate the immune system and to increase peripheral [18, 34, 50] and central [27] cytokines production in murine models. Oral administration of an anorectic DON dose increased IL-1β, IL-6, and TNF-α levels in the hypothalamus and DVC. The concomitant induction of anorexia and up-regulation of pro-inflammatory cytokines within these key structures regulating energy balance may partly explain the DON-induced hypophagia [19, 21, 51]. However, the cell targets of DON within these structures remain to be identified. Given the well-known action of this mycotoxin on innate immune cells such as monocytes and macrophages [52–54], microglial cells, which are the resident macrophages of the CNS, are likely target of DON. Therefore, we first examined the responsiveness of IBA1 in the hypothalamus and brainstem in response to DON administration. IBA1, a 17-kDa actin-binding protein, is specifically and constitutively expressed in microglia [55] and is upregulated during the activation of these cells [56–58]. Therefore, IBA1 is widely used as an immunohistochemical marker for both ramified and activated microglia [57, 59]. IBA1 analysis was performed at 3 and 6 h after DON administration, two time points where anorexia is ongoing. Our main observation was a strong IBA1 up-regulation within the AP, a CVO organ enclosed in the DVC. The CVOs are important brain regions for the initiation of neuroinflammatory responses. Transcriptional activation of genes encoding for pro-inflammatory cytokines occurs most rapidly at the level of the CVOs [60]. Moreover, several lines of evidence support the DVC/AP as a gateway for immune messages from the periphery to higher brain regions. Peripheral inflammation induces robust c-Fos immunoreactivity within the DVC and local cytokine production [61]. The pharmacological inhibition of the DVC prevents activation by i.p. injections of pro-inflammatory molecules into higher brain nuclei, such as the hypothalamus [62]. Within the DVC, a more detailed analysis of microglial morphology revealed an increase in process length and density in response to DON administration. These characteristics are suggestive of the transition from ramified (thin and highly branched processes) to hypertrophic microglia with thick and highly branched processes [3, 63, 64]. Of note, we did not observe an increase in amoeboid microglia in response to DON, whereas when exposed to stress signals such as lipopolysaccharide (LPS) or pro-inflammatory cytokines, microglia transformed from a ramified morphology to an amoeboid form [65, 66]. Within the ME, CVO of the hypothalamus, DON did not induce significant changes in overall IBA1 expression, but quantification of cell body and process length revealed more discreet morphological changes with an increase in process/cell body ratio at 6 h after DON administration. Even if the changes were less marked in the ME, they would still suggest a tendency to microglia hypertrophy. Although the precise functional role of each microglial phenotype is unknown, it has been proposed that the appearance of hypertrophic microglia in the adult brain is linked to the production and release of inflammatory molecules [67–69]. This pattern of microglial cell activation in AP and ME is consistent with works showing microglia activation in these same structures after i.p. injection of LPS [70, 71]. We can assume that circulating DON and blood-borne cytokines reach the AP and ME to activate primarily resident microglia.

### Microglia protects the brain from DON-induced toxicity

This increased microglial reactivity after DON administration in two CVOs led us to test the impact of microglia deletion on DON-induced anorexia. For this purpose, we took advantage of the specific inhibition CSF1R by PLX3397. The activation of CSF1R by its natural ligands (CSF1 and Il-34) is essential for the proliferation, differentiation, and survival of microglia. As a result, CSF1R knockout mice lack microglia [72–74]. In adult mice, treatments with selective CSF1R inhibitors can eliminate up to 99% of the microglial population without inducing behavioral or cognitive abnormalities [32]. As expected, the microglial population in the hypothalamus and DVC decreased by more than 95% after three weeks of treatment with PLX3397. Surprisingly, however, the ablation of microglia enhanced DON's anorectic action to such an extent that a non-toxic dose of DON in control mice became anorectic in PLX3397-treated mice. This was not a result of PLX3397-induced leaky gut since quantification of blood circulating DON after its oral administration showed no difference between control and PLX3397-treated mice. Our result should be compared to data already published using the deletion of microglia in an inflammatory context. Michels et al. [74] reported that microglial cell depletion with clodronate exacerbates both central and systemic inflammation in a model of severe sepsis. Similarly, microglia depletion achieved in C57BL/6 mice by chronic oral administration of PLX5622, a CSF1R antagonist, and in a rat knock-in model did not abrogate LPS-induced expression of pro-inflammatory cytokines in the brain and even worsened it for some of the cytokines. Also, microglia depletion exacerbated LPS-induced sickness activity in mice [75]. Moreover, the inhibition of LPS-induced microglial proliferation with continuous intracerebroventricular infusion of the mitotic inhibitor, cytosine arabinoside, has been reported to prolong LPS-induced sickness responses [76]. Collectively, these results suggest that a transient activation in the microglial population is beneficial during endotoxin-induced inflammation in the rodent brain as it attenuates sickness responses. In this context, our results reinforce the hypothesis that microglia play a protective function during acute inflammation by demonstrating for the first time that it protects against poisoning by a mycotoxin contaminant present in animal and human food.

### Microglial heterogeneity within and around the CVOs

At this point, the question arises of the link between the microglial reactivity that we observed in response to DON in the AP and, to a lesser extent, in the ME, and the exertion of anorectic effects of DON after microglia deletion. In another other words, does the microglial population stimulated by DON become protective? Answering this question is extremely challenging and things are probably even more complicated with the presence of several microglial phenotypes [77]. While microglia are known to respond to different injuries and pathologies with similar morphological alterations [3], it is uncertain whether this will result in similar functional changes. The hypothesis that distinct subtypes of activated microglia characterize specific disease states has been around for a long time. It has long been thought that microglia mostly have pro-inflammatory functions and are therefore considered "bad" for the pathology outcome, whereas alternatively activated "good" microglia were considered important for the anti-inflammatory, scavenging, and regenerative functions that reduce pathology. Nevertheless, this concept of clearly polarized microglia types has been challenged by new research [78, 79]. To characterize more precisely the population of microglia present within the AP and ME, we used, in addition to IBA1, classically phenotypic microglial markers. TMEM119 has been shown to be stably and specifically expressed in microglia, and it can be used to distinguish microglia from resident and infiltrating macrophages [80]. In control animals, TMEM119 labeling revealed the different polarization of microglia cells located within the AP and ME or surrounding integrative nuclei, i.e., NTS and ARC. Further, by using CD11b or CD68 markers, we confirm the existence of several microglial subpopulations in these structures. Microglial CD11b expression has been reported in various neuroinflammatory processes and diseases and increased CD11b expression correlates to the severity of microglial activation [81]. On the other hand, CD68 is a transmembrane lysosomal glycoprotein protein expressed at high levels by macrophages and activated microglia and at low levels by resting microglia. CD68 is a highly upregulated marker of lysosomal activity marker during inflammation which indicates phagocytic activity [82]. CD11b expression was confined to the CVOs organs, CD68 positive cells were mainly located at the interface between the AP and the NTS, i.e., the *funiculus separans*. Similarly, at the hypothalamic level, a subpopulation of microglial cells strongly expressing CD68 was present between the ME and the ARC. We then performed cluster of differentiation cluster 206 (CD206) immunodetection in the hypothalamus and brainstem. CD206, a 175-kDa transmembrane protein encoded by the mannose receptor C-type 1 gene, is mostly expressed in macrophages, dendritic cells, and endothelial cells, where it functions as a receptor for mannosylated ligands. In neural tissues, CD206 expression has also been observed in microglia, where it is widely recognized as a representative anti-inflammatory microglial marker [82]. Accordingly, CD206 is believed to play an important role in the first step of pathogen recognition and uptake in neural tissues [83]. A few IBA1-positive cells were also CD206 positive in control hypothalamus and DVC. These IBA1+/CD206+ cells are localized in the AP and EM. Altogether, these data highlighted the presence, under resting conditions, of different phenotypic microglial subpopulations in the AP and ME and in the areas adjacent to these CVOs.

### Microglia cells around CVOs act as sentinel cells?

The unexpected observation of microglial subpopulations strongly expressing CD68 or CD11b under physiological conditions is puzzling. Their location is intriguing as these cells were found exclusively in the AP and ME and surrounding regions, forming a clear difference between these CVOs and neighboring integrating nuclei such as NTS and ARC. We may presume that these cells are continuously maintained in their functionally activated state under physiologically healthy conditions since they strongly express CD68 or CD11b, two markers found in active but not in resting microglia. We have shown that CD68+ cells were capable of phagocytosis under physiological conditions. Moreover, they could endocytose the peripherally injected FG which enters the brain through the CVOs. It is important to state here that resting microglia have been shown to be unable to uptake FG [84]. These authors reported that injection of FG into adult rat brain resulted in widespread labeling of neurons and perivascular cells, but not endogenous microglial cells, indicating that resting microglia are not active endocytosis cells of the CNS. While the adult brain vasculature has a BBB that prevents water-soluble chemicals from freely entering the brain parenchyma, the CVOs, which lack a BBB, are "brain windows" that facilitate the diffusion of blood-derived chemicals [85]. Based on these results, we propose that this previously undescribed CD68+ and CD11b microglial populations functions as sentinels, limiting the diffusion of endogenous and/or exogenous blood-derived neurotoxic molecules to maintain the parenchymal milieu in structures surrounding CVOs. Takagi et al. [86] discovered a microglial population within CVOs including AP and ME that expressed the active microglia markers CD16/32 and CD86. Taken together, these results paint a complex picture with continuously active microglial subpopulations in and around CVOs that may be responsible for the homeostasis of microenvironments to protect the parenchyma from blood-derived toxic molecules. When these findings are compared to the ablation trials described above, it remains unclear which microglial population deleted by PLX3397 is responsible for the worsening of DON's anorectic effects. If our hypothesis that populations of continuously active microglial cells limit the action of blood-borne, potentially toxic, molecules is correct, inhibition of these cells under physiological conditions should increase their toxicity. Minocycline is commonly used to inhibit microglial activation. Minocycline has been shown in vitro and in vivo to attenuate the induction of pro-inflammatory microglia markers expression during the progressive phase, whereas it does not affect the transient enhancement of anti-inflammatory microglia markers expression in sepsis and amyotrophic lateral sclerosis models, nor microglia stimulation by LPS or interleukin-4 [47, 87]. In our hands, minocycline pretreatment dramatically reduced CD68 expression within the DVC and hypothalamus and worsened DON-induced anorexia and neuronal activation, as we previously observed after microglia deletion by PLX3397. Minocycline, like most antibiotics, has off-targets such as modulation of iron chelation, oxidative stress or leucocyte functions [88, 89], which nevertheless must be kept in mind as interference on the results obtained, even if unlikely. More broadly, even though we used two different pharmacological compounds, i.e., PLX3397 and minocycline, to limit possible non-specific effects, we cannot totally exclude that the modulation of peripheral tissues or cells, such as macrophages contributes, in addition to modulation of microglia activity in CVOs, to exacerbate response to DON. Nevertheless, considering the mechanisms underlying the DON-induced anorexia [22–25], this hypothesis remains speculative.

### DON modified microglia phenotype around CVOs: potential pathological implications

**Fig. 13:**
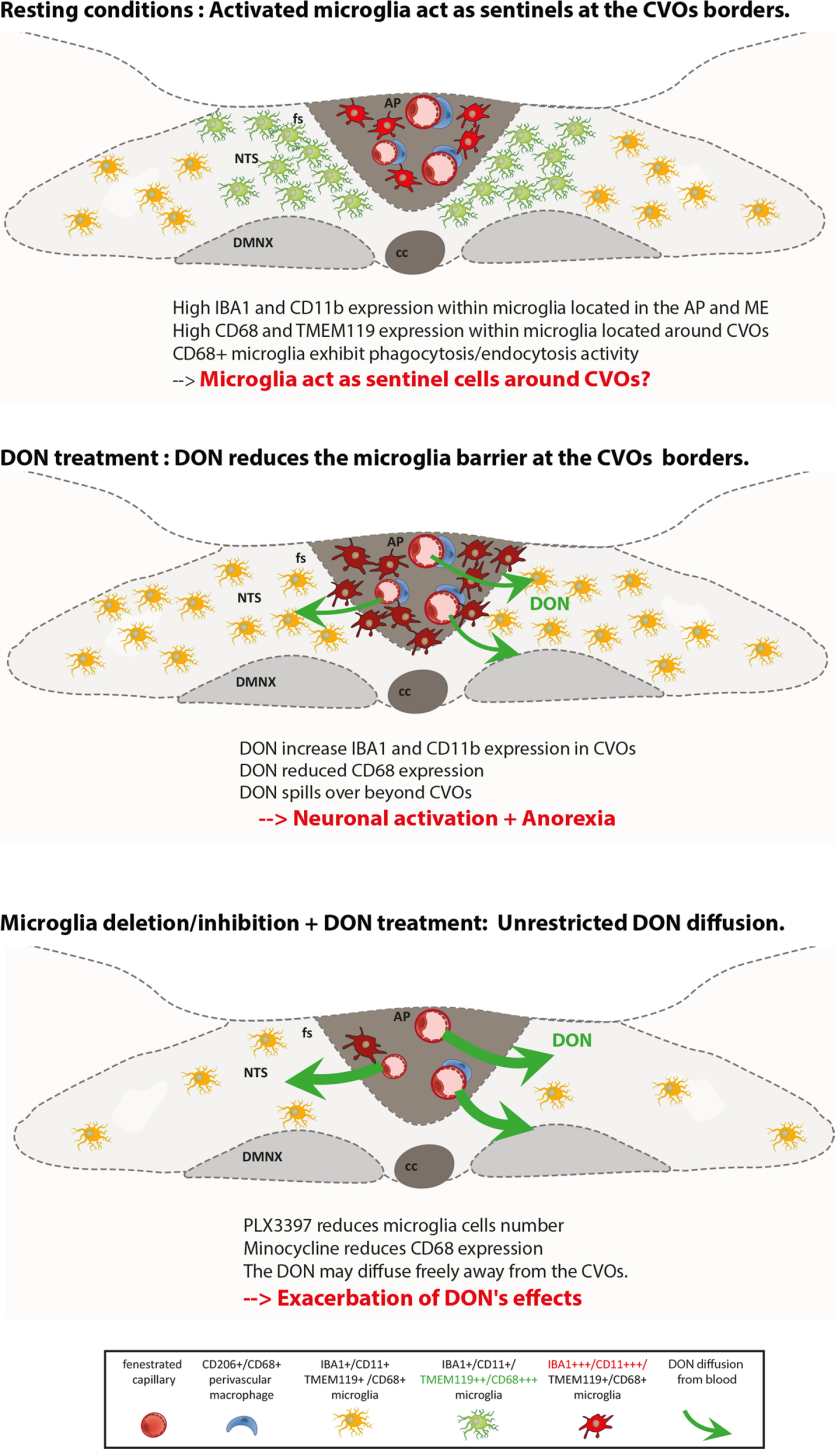
Hypothetical model of the role of microglia in protection against the anorectic effects of DON. Under resting conditions, activated microglial cell populations located at the AP and AP/NTS interface, i.e., the *funiculus separans,* protect the brainstem parenchyma from hazardous molecules coming from the blood. These activated microglia populations may endocyte/phagocytize neurotoxic blood-derived molecules to maintain the parenchymal microenvironment around the CVO. After its administration, DON reaches the AP and diffuses into surrounding structures through fenestrated capillaries. The DON-induced modification of the microglial phenotype could facilitate its action on neuronal networks, responsible for the control of food intake, and located in the NTS. When microglia are deleted by PLX3397 or inhibited by minocycline, the DON diffusion from the AP is unrestricted, leading to an exacerbation of its action

Finally, we also observed that DON altered the microglial cell phenotype not only by increasing IBA1 and CD11b reactivity in the AP and ME, but also decreasing CD68 expression. This last observation is interesting and suggests at least an inhibition of these cells by DON reaching brain parenchyma via CVOs. Our work is the first study evaluating the impact of DON on microglia in vivo*.* Nevertheless, our results are to be compared to two previous studies that evaluated the impact of DON on primary microglial cultures and immortalized human microglial cells [90, 91]. DON has been shown to decrease microglial metabolic activity for 24 h after DON exposure with an IC50 of 7.2 µM [91]. At higher concentrations, DON reduced microglia viability. Microglia were notably more sensitive in the cytotoxicity assay than astrocytes [90, 91]. It is always difficult to extrapolate these results obtained in vitro with in vivo data. The doses of DON in contact with microglial cells are difficult to evaluate in vivo. In addition, in vitro experiments assessed a global DON action regardless of the microglial phenotype. Nevertheless, regarding their possible action as sentinel cells, the modulation of CD68+ and/or CD11b+ microglia could have significant repercussions in physiopathology by reducing the possibility of the organism defense. This could then facilitate the entry into the brain of inflammatory and/or neurotoxic molecules (Fig. 13). The consumption of foods contaminated by this mycotoxin would then have a significant impact on the etiology of neuroinflammatory or neurodegenerative diseases. An obvious limitation of our study that needs to be filled quickly is the lack of information on the kinetics of the reversibility of DON-induced CD68 expression decrease. Several studies have tested the potentiating effect of DON on the toxic/pro-inflammatory effects of LPS [92–95]. Interestingly, mice co-treated with DON 25 mg/kg BW and LPS (1 or 5 mg/kg BW) became moribund before 40 h while DON or LPS administered alone did not induce mortality up to 72 h [92]. Unfortunately, the possibility that this synergy involves a central component has not been investigated here. Yet, it seems important in the near future to test the response to neurotoxic substances of animals having previously received the mycotoxin.

In conclusion, our work showed that DON induced microglial activation in CVOs, but this microgliosis does not appear to contribute significantly to DON toxicity since the deletion or inhibition of microglia exacerbates the DON’s anorectic effect. Unexpectedly, this study also allowed us to highlight the presence of a continuously active microglial subpopulation around CVOs whose role should be further investigated in the coming years. The datasets supporting the conclusions of this article are included within the article and its additional files.

## Supplementary Information

**Additional file 1:** **Figure S1.** Analysis method used to quantify microglial IBA1 staining. **A.** The unprocessed IBA1 picture obtained from a brainstem section. **B.** Definition of the region of interest (ROI). The AP has been selected in the given example. **C. **Brightness and contrast were adjusted to allow full observation of microglia processes. **D. **Image after applying thresholding.** E.** Pixel-clusters that are above an applied staining threshold and size filter are plotted (blacklines) to determine the total cell area of all microglia. Scale bar: 100 µm.AP: area postrema; cc: central canal: XII: hypoglossal nucleus; NTS: nucleus tractus solitarius.

**Additional file 2:** **Figure S2. **Effect of DON administration on GFAP immunoreactivity within the hypothalamus and brainstem**. A-B. **Representative images of GFAP-immunoreactivity in the DVC (A) and hypothalamus (B) of control and DON-treated animals. Images originated from animals sacrificed 3 and 6 h after treatment. Scale bar: 500 μm. **C-D. **Quantitative analysis of GFAP immunoreactivity performed 3 and 6 h afterDON treatment in DVC (C) and hypothalamus (D) of control and DON-treated animals (12.5 mg/kg BW). One way ANOVAns: non significantly different. AP: area postrema; ARC: arcuate nucleus; cc: central canal; ME: median eminence; NTS: nucleus tractus solitarius; 3V: Third ventricle.

**Additional file 3:** **Figure S3. **PLX3397-induced DON hypersensitivity did not result from an increased intestinal absorption.** A. **Plasmatic DON concentration measured 40 min after treatment in control or PLX3397-treated mice having received *p.o.* administration of vehicle (H_2_O) orDON 1.25 mg/kg BW. Two-way ANOVA, *: significantly different from respective control, ***p < 0.001.ns:non significantly different. **B.** Cumulative food intake (g), measuredover a 3 and 6 h periods, of control or PLX3397-treated mice having received administration of either *p.o.* or *i.p.* DON 1.25 mg/kg BW. Two-way ANOVA, *: significantly different from respective control, **p < 0.01, ***p < 0.001.

**Additional file 4:** **FigureS4. **Effect of DON administration on CD206 microglial expression.** A. **Representative photomicrographs of CD206 and IBA1 labeling on brainstem coronal sections fromvehicle-treated and DON-treated mice. Scale bar: 100 µm. **B. **Representative high magnification images of double IBA1/CD206 labeling highlighting the microglial cell phenotype within the AP of vehicle-treated and DON-treatedmice. The white boxes in A indicated regions where panel B originated.Scale bar: 10 µm. **C-D.** Quantification within the AP of the number of CD206+cells (C) and the percentage of CD206+/IBA1+ cells out of the total number of IBA1+cells (D) in control or DON-treated animals.  ns: non significantly different. AP: area postrema; cc: canal central; fs: *funiculus separens;* NTS: nucleus tractus solitarius.

**Additional file 5:** **Figure S5. **Hypothalamic CD68+ microglial cells exhibit endocytic activity at resting conditions.** A.** Low magnification photomicrographs of FG fluorescence and CD68 labeling observedat the hypothalamic level. The animals were sacrificed 3 h after the i.p. FG injection. Note the intense FG signal within the ME illustrating its diffusion from fenestrated capillaries. Scale bar: 500 µm. **B. **IMARIS bitplane images of fluorogold and CD68 co-localization allowing visualization of internalized FG. Scale bar: 500 µm. **C.** High magnification photomicrographs of FG fluorescence and CD68 labelingobserved at the ME level. Scale bar: 200 µm. **D. **IMARIS bitplane images of fluorogold and CD68 co-localization allowing visualization of internalized FG in the ME. Scale bar: 200 µm. ARC: arcuate nucleus; ME: median eminence; 3V: Third ventricle.

**Additional file 6: TableS1. **Immunohistochemistry conditions.

## Data Availability

The data sets used and/or analyzed during the current study are available from the corresponding author on reasonable request.
