# Supplementary figures and images for: Constitutively active microglial populations limit anorexia induced by the food contaminant deoxynivalenol
Source: J Neuroinflammation. 2022 Nov 19;19:280. doi: 10.1186/s12974-022-02631-7 (PMC9675145; doi:10.1186/s12974-022-02631-7)

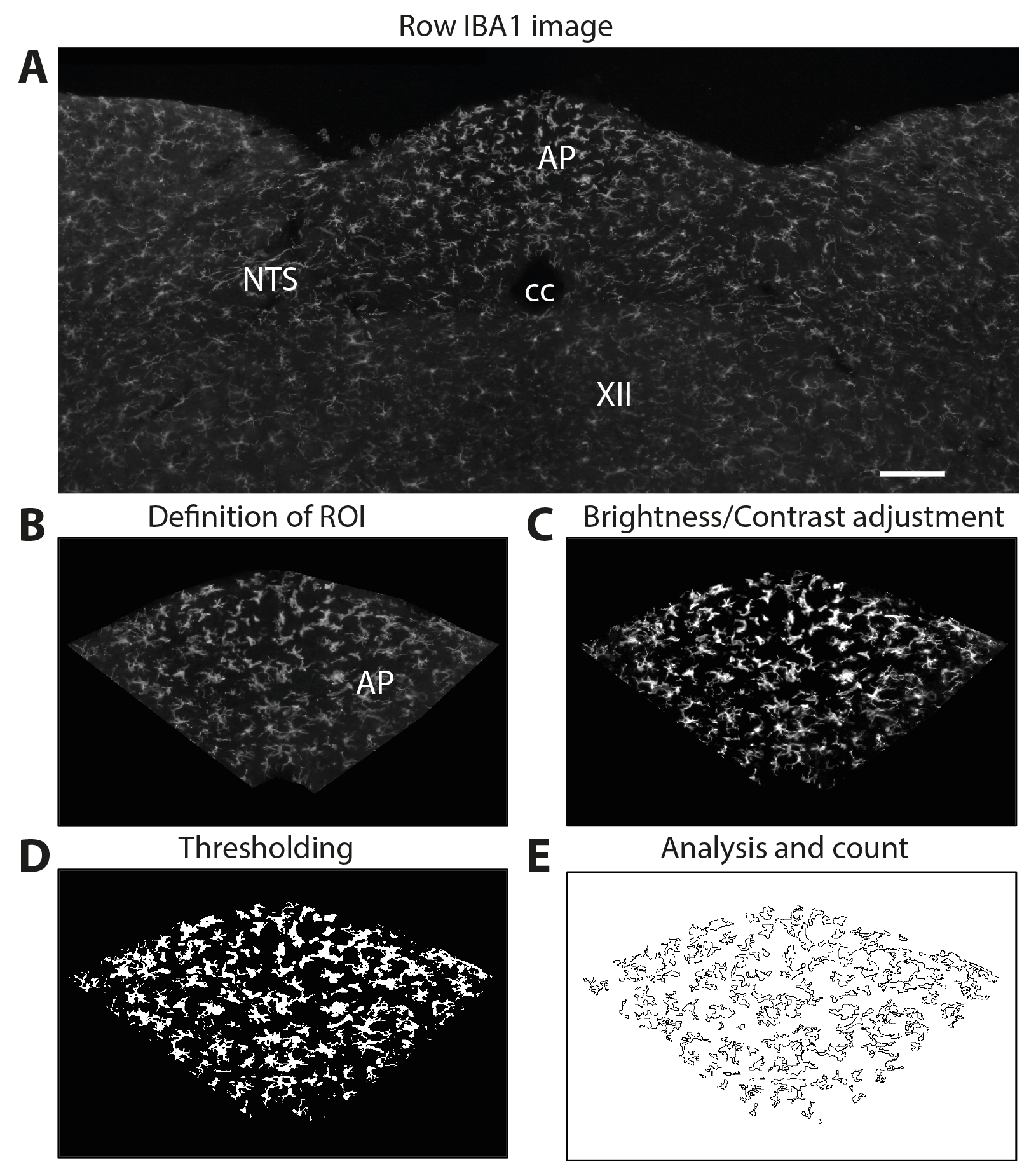

Supplement: Supplementary file 1 — Additional file 1: Figure S1. Analysis method used to quantify microglial IBA1 staining. A. The unprocessed IBA1 picture obtained from a brainstem section. B. Definition of the region of interest (ROI). The AP has been selected in the given example. C. Brightness and contrast were adjusted to allow full observation of microglia processes. D. Image after applying thresholding. E. Pixel-clusters that are above an applied staining threshold and size filter are plotted (blacklines) to determine the total cell area of all microglia. Scale bar: 100 µm.AP: area postrema; cc: central canal: XII: hypoglossal nucleus; NTS: nucleus tractus solitarius. [file 12974_2022_2631_MOESM1_ESM.tif]

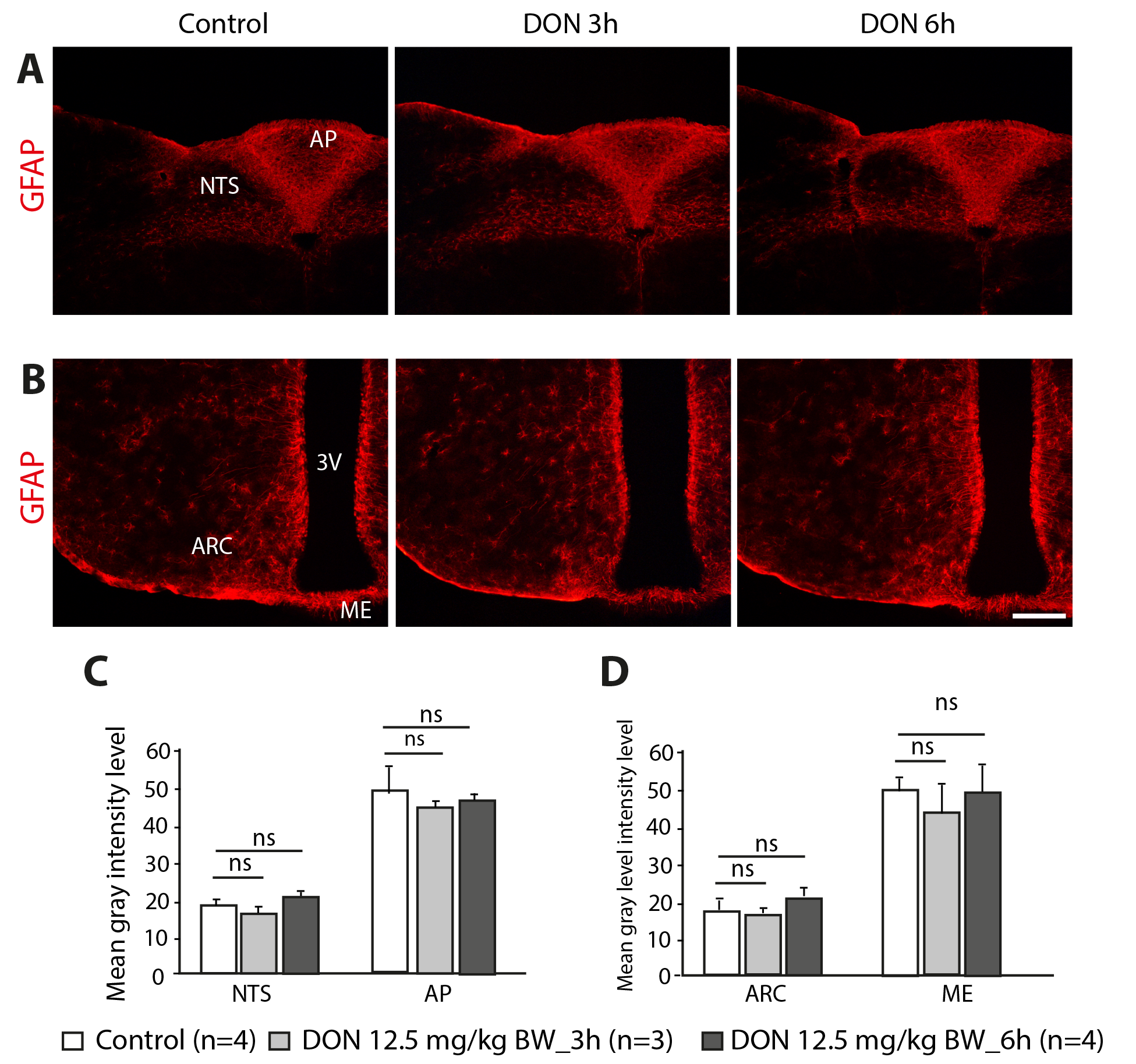

Supplement: Supplementary file 2 — Additional file 2: Figure S2. Effect of DON administration on GFAP immunoreactivity within the hypothalamus and brainstem. A-B. Representative images of GFAP-immunoreactivity in the DVC (A) and hypothalamus (B) of control and DON-treated animals. Images originated from animals sacrificed 3 and 6 h after treatment. Scale bar: 500 μm. C-D. Quantitative analysis of GFAP immunoreactivity performed 3 and 6 h afterDON treatment in DVC (C) and hypothalamus (D) of control and DON-treated animals (12.5 mg/kg BW). One way ANOVAns: non significantly different. AP: area postrema; ARC: arcuate nucleus; cc: central canal; ME: median eminence; NTS: nucleus tractus solitarius; 3V: Third ventricle. [file 12974_2022_2631_MOESM2_ESM.tif]

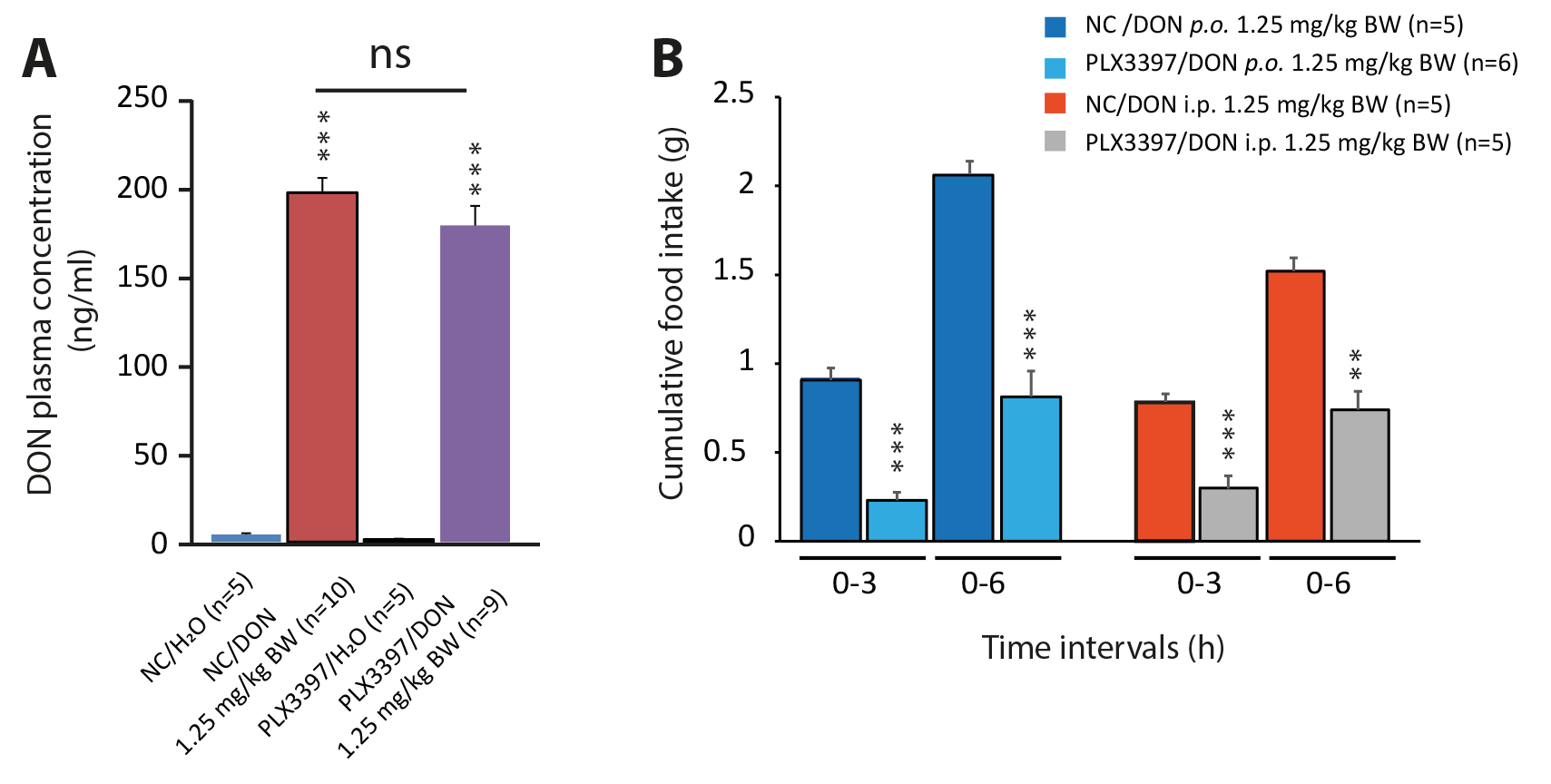

Supplement: Supplementary file 3 — Additional file 3: Figure S3. PLX3397-induced DON hypersensitivity did not result from an increased intestinal absorption. A. Plasmatic DON concentration measured 40 min after treatment in control or PLX3397-treated mice having received p.o. administration of vehicle (H2O) orDON 1.25 mg/kg BW. Two-way ANOVA, *: significantly different from respective control, ***p < 0.001.ns:non significantly different. B. Cumulative food intake (g), measuredover a 3 and 6 h periods, of control or PLX3397-treated mice having received administration of either p.o. or i.p. DON 1.25 mg/kg BW. Two-way ANOVA, *: significantly different from respective control, **p < 0.01, ***p < 0.001. [file 12974_2022_2631_MOESM3_ESM.tif]

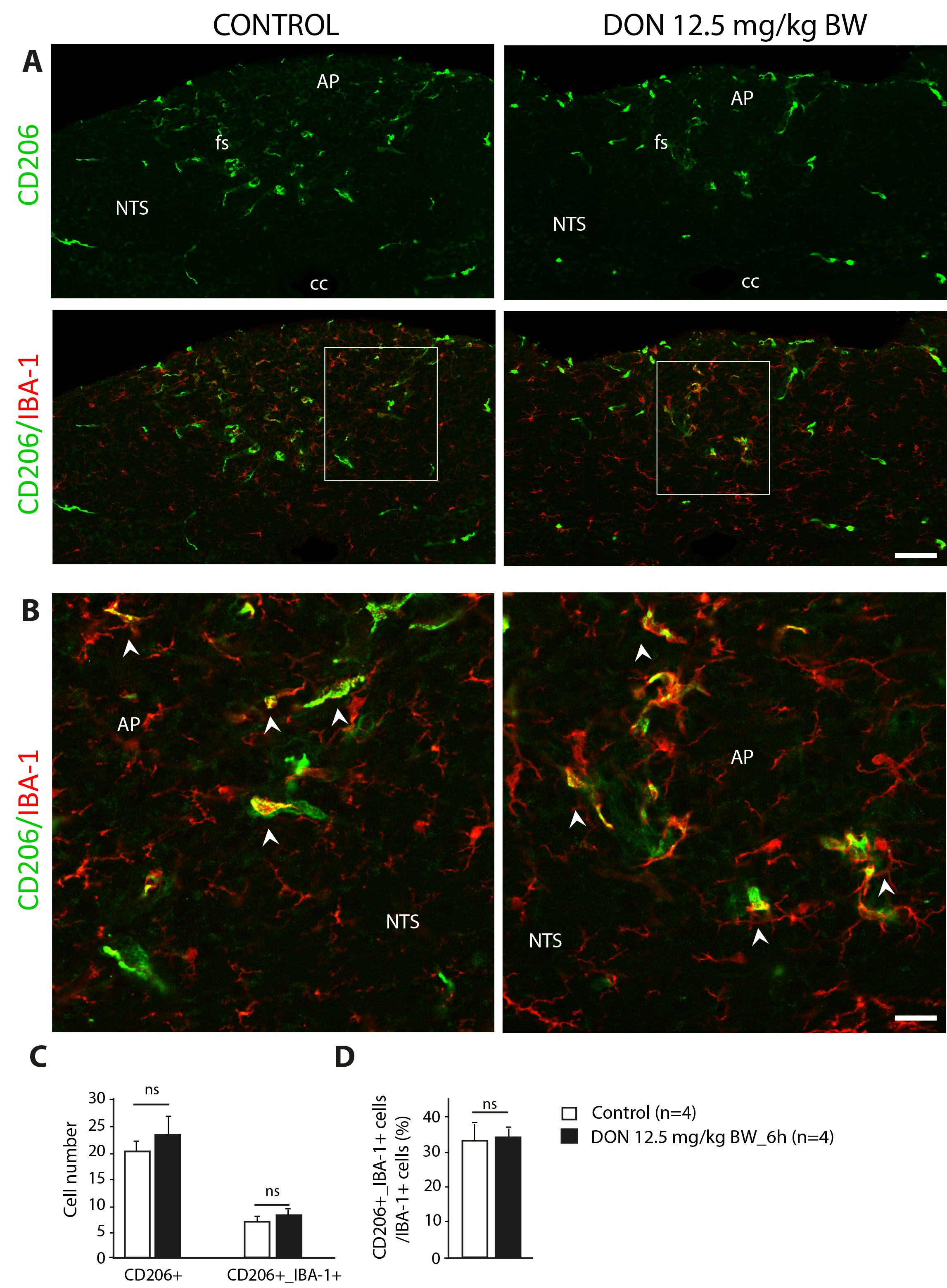

Supplement: Supplementary file 4 — Additional file 4: FigureS4. Effect of DON administration on CD206 microglial expression. A. Representative photomicrographs of CD206 and IBA1 labeling on brainstem coronal sections fromvehicle-treated and DON-treated mice. Scale bar: 100 µm. B. Representative high magnification images of double IBA1/CD206 labeling highlighting the microglial cell phenotype within the AP of vehicle-treated and DON-treatedmice. The white boxes in A indicated regions where panel B originated.Scale bar: 10 µm. C-D. Quantification within the AP of the number of CD206+cells (C) and the percentage of CD206+/IBA1+ cells out of the total number of IBA1+cells (D) in control or DON-treated animals. ns: non significantly different. AP: area postrema; cc: canal central; fs: funiculus separens; NTS: nucleus tractus solitarius. [file 12974_2022_2631_MOESM4_ESM.tif]

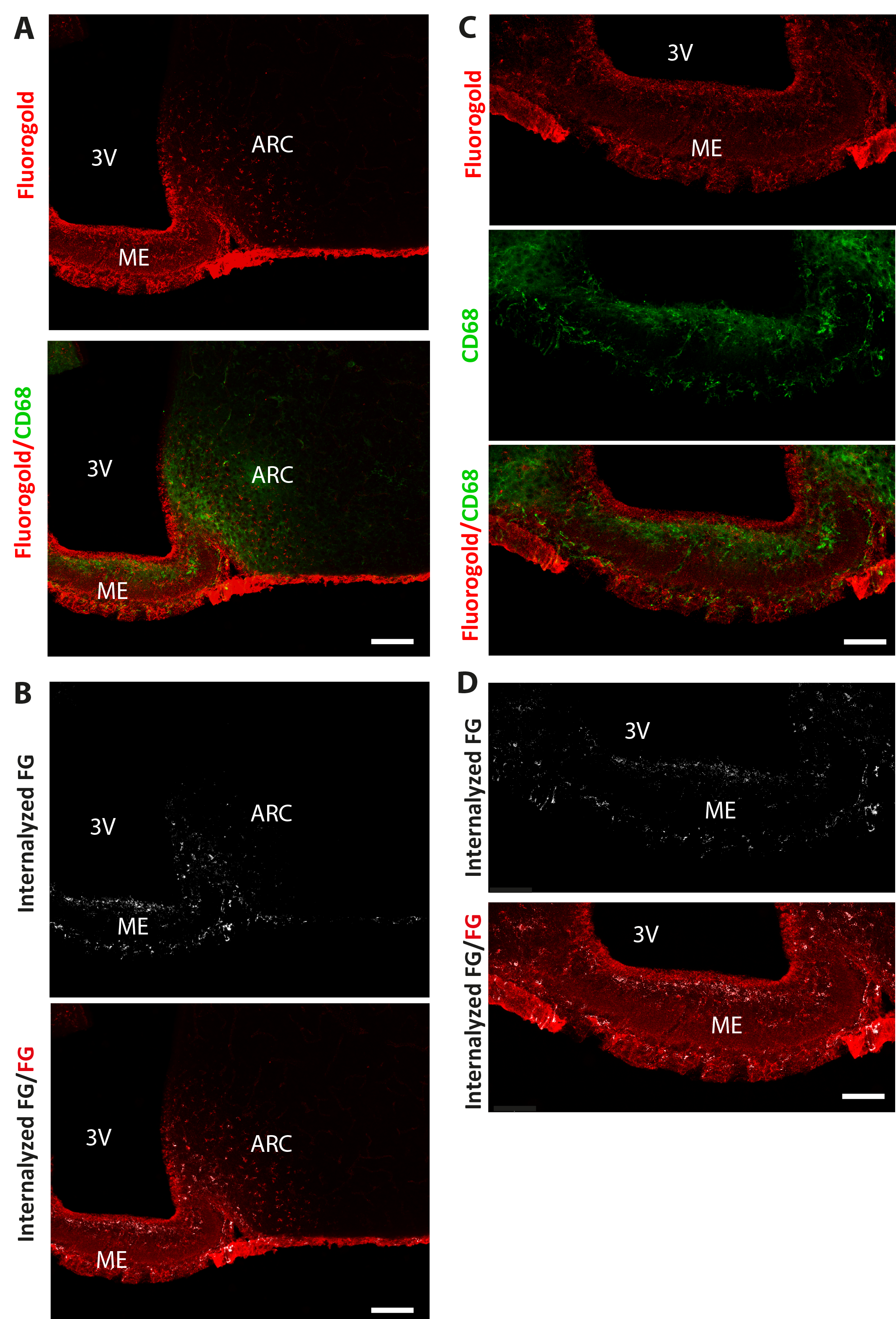

Supplement: Supplementary file 5 — Additional file 5: Figure S5. Hypothalamic CD68+ microglial cells exhibit endocytic activity at resting conditions. A. Low magnification photomicrographs of FG fluorescence and CD68 labeling observedat the hypothalamic level. The animals were sacrificed 3 h after the i.p. FG injection. Note the intense FG signal within the ME illustrating its diffusion from fenestrated capillaries. Scale bar: 500 µm. B. IMARIS bitplane images of fluorogold and CD68 co-localization allowing visualization of internalized FG. Scale bar: 500 µm. C. High magnification photomicrographs of FG fluorescence and CD68 labelingobserved at the ME level. Scale bar: 200 µm. D. IMARIS bitplane images of fluorogold and CD68 co-localization allowing visualization of internalized FG in the ME. Scale bar: 200 µm. ARC: arcuate nucleus; ME: median eminence; 3V: Third ventricle. [file 12974_2022_2631_MOESM5_ESM.tif]
