# Supplementary material for: Constitutively active microglial populations limit anorexia induced by the food contaminant deoxynivalenol
Source: J Neuroinflammation. 2022 Nov 19;19:280. doi: 10.1186/s12974-022-02631-7 (PMC9675145; doi:10.1186/s12974-022-02631-7)
Supplement: Supplementary file 6 — Additional file 6: TableS1. Immunohistochemistry conditions. [file 12974_2022_2631_MOESM6_ESM.docx]

| **Immunostaining** | **Primary antibody** | **Secondary antibody** | **Serum blocking** |
| --- | --- | --- | --- |
| IBA-1 | Goat polyclonal anti-IBA1 (1:500) ab5076, Abcam | Donkey anti-Goat Alexa Fluor 488 (1:400)  A11055, Invitrogen | Horse serum 3% |
| c-Fos | Rabbit polyclonal anti-c-Fos (1:3000)  ABE457, Merk Millipore | Biotinylated Goat anti-Rabbit (1:400)  BA-1000, Vector Labs | Normal Goat serum 3% |
| IBA-1/CD68 | -Rabbit polyclonal anti-IBA1 (1:4000)  019-19741, Fujifilm Wako  -Mouse monoclonal anti-CD68 (1:200)  E-AB-22013, Elabscience | -Donkey anti-Rabbit Alexa Fluor 594 (1:400)  A21207, Invitrogen  -Goat anti-Mouse Alexa Fluor 488 (1:400)  A11029, Invitrogen | -Bovine serum albumine 3%  -Bovine serum albumine 3% |
| IBA-1/CD206 | -Rabbit polyclonal anti-IBA1 (1:4000)  019-19741, Fujifilm Wako  -Goat polyclonal anti-CD206 (1:500)  AF2535, R&D Systems | -Donkey anti-Rabbit Alexa Fluor 594 (1:400)  A21207, Invitrogen  -Donkey anti-Goat Alexa Fluor 488 (1:400)  (A11055, Invitrogen) | -Bovine serum albumine 3%  -Bovine serum albumine 3% |
| IBA-1/CD11b | -Rabbit polyclonal anti-IBA1 (1:4000)  019-19741, Fujifilm Wako  -Rat monoclonal anti-CD11b (1:100)  Sc-23937, Santa Cruz Biotechnology | -Donkey anti-Rabbit Alexa Fluor 488 (1:400)  A21206, Invitrogen  -Goat anti-Rat Alexa Fluor 594 (1:400)  A11007, Invitrogen | -Bovine serum albumine 3%  -Bovine serum albumine 3% |
| CD11b/CD68 | -Rat monoclonal anti-CD11b (1:100)  Sc-23937, Santa Cruz Biotechnology  -Mouse monoclonal anti-CD68 (1:200)  E-AB-22013, Elabscience | -Goat anti-Rat Alexa Fluor 594 (1:400)  A11007, Invitrogen  -Goat anti-Mouse Alexa Fluor 488 (1:400)  A11029, Invitrogen | -Bovine serum albumine 3%  -Bovine serum albumine 3% |
| IBA-1/TMEM119 | -Goat polyclonal anti-IBA1 (1:500) ab5076, Abcam  -Rabbit polyclonal anti-TMEM119 (1:2000) GTX134087, GeneTex | -Donkey anti-Goat Alexa Fluor 488 (1:400)  A11055, Invitrogen  -Donkey anti-Rabbit Alexa Fluor 594 (1:400)  A21207, Invitrogen | -Horse serum 3%  -Bovine serum albumine 3% |
| TMEM119/CD68 | -Rabbit polyclonal anti-TMEM119 (1:2000) GTX134087, GeneTex  -Mouse monoclonal anti-CD68 (1:200)  E-AB-22013, Elabscience | -Donkey anti-Rabbit Alexa Fluor 594 (1:400)  A21207, Invitrogen  -Goat anti-Mouse Alexa Fluor 488 (1:400)  A11029, Invitrogen | -Bovine serum albumine 3%  -Bovine serum albumine 3% |
| FG/CD68 | -Rabbit polyclonal anti-FG (1:1000) AB153, Chemicon  -Mouse monoclonal anti-CD68 (1:200)  E-AB-22013, Elabscience | -Donkey anti-Rabbit Alexa Fluor 594 (1:400)  A21207, Invitrogen  -Goat anti-Mouse Alexa Fluor 488 (1:400)  A11029, Invitrogen | -Normal Goat serum 3%  -Bovine serum albumine 3% |
| FG/IBA-1 | -Rabbit polyclonal anti-FG (1:1000) AB153, Chemicon  Goat polyclonal anti-IBA1 (1:500) ab5076, Abcam | -Donkey anti-Rabbit Alexa Fluor 594 (1:400)  A21207, Invitrogen  Donkey anti-Goat Alexa Fluor 488 (1:400)  (A11055, Invitrogen) | -Normal Goat serum 3%  -Horse serum 3% |

**Table S1:** Immunohistochemistry conditions
